# Perinatal mental health and its social determinants: qualitative findings from rural Telangana

**DOI:** 10.1186/s12888-026-08066-1

**Published:** 2026-05-23

**Authors:** Annie Wang, Sreya Majumdar, Sudhir Raj Thout, Josyula K. Lakshmi, Jane Hirst, Pallab K. Maulik, Devarsetty Praveen, Nicole Votruba

**Affiliations:** 1 Nuffield Department of Women’s and Reproductive Health, University of Oxford, Oxford, UK; 2 The George Institute for Global Health, New Delhi, India; 3 PHFI Institute of Public Health Sciences, Hyderabad, India; 4 The George Institute for Global Health UK, at Imperial College London, London, UK; 5 University of New South Wales, Sydney, Australia; 6 The George Institute for Global Health, Sydney, Australia

**Keywords:** Perinatal mental health, Social determinants, Perinatal common mental disorders, Telangana, South India, Socio-ecological model

## Abstract

**Background:**

The prevalence and severity of perinatal common mental disorders (CMDs) are disproportionately high in India and other low- and middle- income contexts. Since social determinants strongly influence perinatal CMDs, they represent promising targets for alleviating the burden of disease in these countries. This study aimed to understand (1) social determinants of perinatal mental health and their concepts, context, interactions, facilitators, and impact and (2) how interventions targeting these determinants should be designed and implemented in Telangana and similar regions across rural South India.

**Methods:**

Nine focus group discussions and eight individual interviews were conducted to capture perspectives from a total of 65 stakeholders, including community health workers, women at risk for perinatal CMDs, their caregivers, treatment providers, and village leaders in eight villages across the Siddipet district of Telangana, India. Social determinants were derived from the resulting narratives through a thematic analysis with a hybrid inductive and deductive approach.

**Results:**

Thirty-six social determinants of perinatal mental health were identified and mapped onto the six levels of influence (individual, interpersonal relationships, organizations, community, policy, society) within the socio-ecological model of perinatal mental health. Across these six levels, five themes regarding these social determinants and their relationships, context, and influence on perinatal mental health in Telangana emerged: attitudes about pregnancy outcomes, gender inequity, relationships and support, mental health stigma, and mental healthcare access. Considerations for perinatal CMD interventions that participants proposed are also presented, along with our practical suggestions to address them.

**Conclusions:**

A wide range of social determinants impact perinatal mental health within Telangana. Since these factors often have impacts across multiple levels of influence, interventions should employ a multi-faceted, intersectoral approach to effectively prevent, recognize, and treat perinatal CMDs in Telangana and similar regions across rural South India.

**Clinical trial number:**

Not applicable.

**Supplementary Information:**

The online version contains supplementary material available at 10.1186/s12888-026-08066-1.

## Background

Physiological, social, and behavioral changes during the perinatal period (pregnancy and the first year after childbirth) increase women’s risk for mental health problems [1, 2]. Perinatal mental health conditions affect approximately 10% of pregnant women and 13% of postnatal women worldwide [3]. In low- and middle- income countries like India, these rates are even higher, with an estimated 16% of pregnant women and 20% of postnatal women experiencing a mental health issue [2]. This difference is attributed to substantially elevated rates of perinatal common mental disorders (CMDs) such as depression and anxiety in low- and middle-income settings [4, 5]. Studies on perinatal CMDs from South India are rare [6–9], however, a recent study from Telangana found a significant prevalence of postnatal depression (25%), anxiety (25%), and both (11%) [10]. This is consistent with a meta-analysis that estimated the prevalence of postnatal depression in South India to be 26% (95% CI: 19–32), the highest in the country [11]. Studies have also indicated a significant prevalence of antenatal depression (16–22%), anxiety (23%), and both (13%) in South India [12, 13].

Perinatal mental health conditions have been linked to poor maternal well-being, behavior, and functioning; women with perinatal CMDs are at increased risk for gestational hypertension [14], pre-eclampsia [15], preterm birth [16], miscarriage [17], diabetes [18], cardiovascular disease [19], and suicide [20, 21]. Poor perinatal mental health can also disrupt mother-child bonding [22] and breastfeeding practices [23, 24].

Additionally, perinatal CMDs are associated with adverse effects on the fetus in-utero, including fetal growth restrictions, low birthweight, small size for gestational age [16], and after birth, with increased incidence of diarrheal diseases [25], and impaired social, behavioral, and cognitive development [26–29]. These conditions also increase the child’s risk for mental disorders [30, 31] and suicidality [32] through adolescence.

Social determinants are non-medical factors that influence health outcomes, including the conditions in which people are born, grow, work, live, and age [33]. The influence of social determinants on mental health outcomes has been established in recent years [34–36], with a recent study suggesting that they account for approximately 60–90% of mental illnesses [37]. These findings illustrate that medical treatments alone cannot adequately relieve the global burden of mental illnesses; complementary interventions that address socio-economic context and influences are needed [36, 38–40].

Developing interventions to address social determinants of mental health necessitates in-depth understandings of these factors, including their influence on perinatal mental health and interactions in specific settings and populations [41]. While studies have identified social support [42], material hardship [43], low income [44], language barriers [45], distance to healthcare [46], and domestic violence [47] as key social determinants of perinatal mental health, most of these investigations to date have focused on high income countries [48]. Prior research in India is limited to a handful of studies that mostly assess selected determinants [49, 50], singular mental health issues [51, 52], and/or specific phases of the perinatal period [12, 13, 53]. Further, most studies have focused on evaluating correlations between these factors and the risk, prevalence, and severity of perinatal CMDs, rather than understanding the socio-cultural norms and structures that drive these interactions [54–56]. The two publications [57, 58] that have captured a broad range of contexts and concepts relevant to social determinants of perinatal mental health disorders in India presented insights from North and South India together. As there are considerable differences in language, religion, culture [59], and reproductive outcomes and behavior [60, 61] between these two regions, we currently lack the comprehensive understanding of the social determinants of perinatal mental health that is needed to guide the development of effective community-based interventions specific to the rural South Indian context.

This study aimed to identify and understand key social determinants of perinatal mental health in a rural South Indian context, including their concepts, relationships, facilitators, and impact, by examining narratives from rural villages in Telangana, India. A secondary aim was to understand how a perinatal mental health intervention could be designed and implemented to address the identified social determinants in a manner that is appropriate, acceptable, and feasible in these communities. This research is part of the PeRinAtal Mental Health (PRAMH) study that is being conducted across rural areas in Haryana and Telangana, India [62].

## Methods

### Study design

This qualitative study was conducted to understand how perinatal mental health is experienced and perceived by communities in rural Telangana and elucidate their recommendations for future mental health interventions.

### Study context

This study was conducted in 8 rural villages in the Siddipet district of Telangana, India where the PRAMH study team had established relationships with local village communities, health care workers, primary healthcare staff, and district health officials. Telangana is a state in South India that was established in 2014, with a majority (60%) of its 35 million inhabitants living in rural areas [63] and practicing Hinduism.

### Participants and recruitment

To capture a breadth of perspectives, the researchers utilized a holistic approach to identify key stakeholders from the healthcare system, care services, and community support organizations whose roles and responsibilities influenced women’s experiences with perinatal CMDs in the region, and invited these stakeholders to participate in this study from September to November 2022 through phone calls and *via* WhatsApp messaging. These included Accredited Social Health Activists (ASHAs), Auxiliary Nurse Midwives (ANMs), Anganwadi workers (AWWs), mental health specialists, AYUSH (Ayurveda, Yoga & Naturopathy, Unani, Siddha, and Homeopathy) doctors, and maternal healthcare providers from primary and tertiary healthcare centers in the Siddipet district as well as self-help group leaders and district health coordinators and officers. Local ASHAs and ANMs also purposively sampled for women at risk for perinatal CMDs at local antenatal clinics by approaching and recruiting those that appeared in distress, or who faced challenges (including but not limited to infant mortality, single/widowed mothers, birth of female child, physical health conditions, and induced or spontaneous abortion) during current or recent pregnancies. At-risk women that were between 12 and 36 weeks of gestation or within 6 months postpartum at the time of recruitment and were planning to stay in the village after childbirth were invited by these community health workers (CHWs) to participate in the study along with their families/caregivers. Researchers monitored the CHW-mediated recruitment process to reduce the potential for gatekeeper and sampling bias. Individuals across all stakeholder groups that declined participation, did not speak and understand verbal English, Hindi, or Telugu (the official language in Telangana), were unable to give written consent for participation, and were under 18 years old at the time of recruitment were excluded. All participants were informed about the study aims and data collection process and gave written consent for their participation prior to their interviews. Participant recruitment continued until the researchers determined that adequate data saturation had been achieved and no novel insights emerged from the interviews.

### Data collection

Nine focus-group discussions (FGDs) and eight in-depth interviews (IDIs) were conducted between September to November 2022, capturing insights from 65 participants (Table 1). Women at risk for perinatal CMDs (*n* = 17), families/caregivers (*n* = 16), AWWs (*n* = 13), ASHAs (*n* = 10), and ANMs (*n* = 1) attended separate in-person FGD sessions at local village primary health centers, sub-centers, and AWW centers. Each FGD lasted approximately 90 min and was led by the third and fourth authors in Telugu with support from the last author, local female facilitators, and two male field staff members. IDIs were conducted either in-person, over the phone, or through virtual platforms (Microsoft Teams, Skype). Each IDI lasted approximately one hour and was conducted by either the last author in English or the local project manager in Telugu. All interviews followed topic guides [see Additional file 1] informed by Kleinman, et al. [64] and were audio recorded, transcribed verbatim, translated to English, and anonymized prior to analysis to protect participant privacy. Project leads and interviewers underwent training on mental health and stigma prior to the interviews and made field notes during interviews to support the analysis process. Participants were informed of the interviewers’ independent researcher roles and reassured that their identities would be kept private before their interviews began, and sessions were conducted in a manner that maximized respect, reflexivity, and participant comfort. Researchers paid attention to language use, tone, and non-verbal communication to foster rapport and mitigate power dynamics arising from interviewer positionalities, outsider statuses, and gender differences, especially during FGDs.

**Table 1:** Overview of data collection

Interview type	Interview number	Participant type	No. of participants
Focus group discussion (FGD)	FGD1	AWWs	4
	FGD2	AWWs (4), ASHAs (2)	6
	FGD3	AWWs	5
	FGD4	ASHAs	4
	FGD5	ASHAs (4), ANM (1)	5
	FGD6	Caregivers	7
	FGD7	Caregivers	9
	FGD8	Women at risk for perinatal CMDs	6
	FGD9	Women at risk for perinatal CMDs	11
In-depth interview (IDI)	IDI1	Clinician	1 per IDI
	IDI2	Mental health professional	
	IDI3	AYUSH (Ayurveda, Yoga & Naturopathy, Unani, Siddha, and Homeopathy) doctor	
	IDI4	Private gynecologist	
	IDI5	Self-help group leader	
	IDI6	General practitioner	
	IDI7	Medical officer	
	IDI8	Psychiatrist	
Total interviews	**17**	**Total participants**	**65**

AWW = Anganwadi worker, ASHA = Accredited Social Health Activist, ANM = Auxiliary Nurse Midwife

### Analysis

Following Braun and Clarke [65] approach, the authors conducted a thematic analysis of the data in five stages: (i) familiarization with the data, (ii) generating initial codes, (iii) searching for themes, (iv) reviewing themes, and (v) defining and naming themes. In the first stage, three researchers (AW, NV, SM) completed a readthrough of the dataset before developing an initial set of codes denoting recurring topics that participants discussed in relation to perinatal mental health (e.g., the ‘understandings of mental health’ code was used to label areas in transcripts wherein participants detailed their own or common perceptions of mental health). The first author (AW) then organized these initial codes into six categories corresponding to the six levels of influence in Michaels, et al. [66] Mental Health and Well-being Ecological model. Additional codes informed by social determinants of perinatal mental health identified by Fisher, et al. [4] and Varma, et al. [67] were added. Six additional codes were also created to capture participants’ views on future interventions and were organized into a seventh category. This coding framework was first applied by AW to three transcripts to assess its ability to capture participant experiences and understandings of perinatal CMDs. Afterwards, AW and the last author (NV) reviewed the initial coding of these transcripts to ensure that the codebook was applied and understood consistently and provided sufficient coverage of topics across interviews. Codes were added, removed, refined, and combined accordingly before AW applied the final framework (Table 2) containing 53 total codes to all transcripts using the NVivo 14 software program.

**Table 2:** The final coding framework for social determinants of perinatal mental health

Category	Codes
**Individual**: Biological and personal characteristics of an individual	Adverse pregnancy outcome; educational status, fear of pregnancy outcome; fertility; internal pregnancy expectations; maternal age; maternal health; number of pregnancies; social class
**Interpersonal relationships**: The individual’s formal and informal social networks that influence their behavior and contribute to their experiences	Attachment to child; caring for child; external pregnancy expectations; familial abuse and neglect; infant gender; infant health; intimate partner violence; planning of pregnancy; previous female child; previous male child; quality of relationship with husband; quality of relationship with in-laws; quality of relationship with own family; support during perinatal period
**Organizations**: Influence of public, private, and non-profit organizations and institutions on health	Healthcare accessibility; healthcare availability and quality; current mental healthcare and treatments; patient-provider relationships; provider education and knowledge; provider relationships with family members
**Community**: Characteristics of settings in which people have social relationships	Awareness of mental healthcare; community relationships; diet quality; employment conditions; employment status and housework; finances; food security and intake; housing conditions
**Policy**: Laws and policies that regulate and support health behaviors	Governmental and policy support
**Society**: Broad factors that favor or impair health at the societal level	Alcohol use; cultural and faith-based practices; cultural acceptance of violence; discrimination and exclusion; expectations of women; female autonomy, empowerment, and agency; mental health stigma; stigma about women; understandings of mental health
**Future intervention**: Suggestions and considerations for the development of future perinatal mental health interventions	Recommendations at individual level; recommendations at interpersonal relationships level; recommendations at organizations level; recommendations at community level; recommendations at policy level; recommendations at societal level

**Fig. 1:**
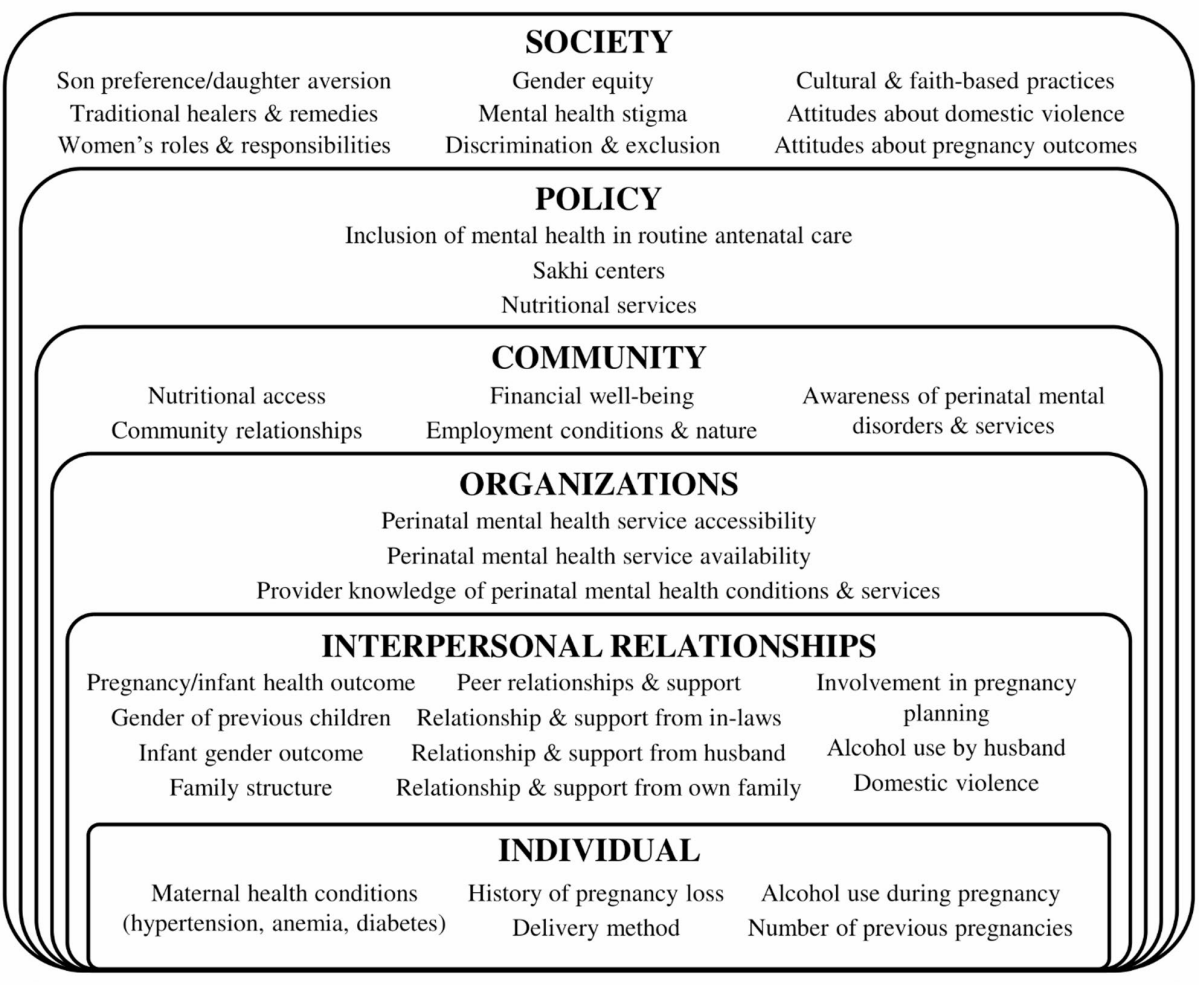
Socio-ecological Model of Perinatal Mental Health. This conceptual framework describes 36 social determinants of perinatal common mental health in rural Telangana. Adapted from Michaels, et al. (2022)'s Mental Health and Well-being Ecological Model [66]

Once the coding was completed, AW and NV reviewed the transcript excerpts that had been assigned to each code to determine whether and how the social, structural, or contextual factors they represented were connected to perinatal mental health. The factors that were determined to directly influence perinatal mental health outcomes, including as participant-identified stressors or barriers to care, became denoted as social determinants. Codes containing similar or complementary insights from participants, such as ‘previous female child’ and ‘previous male child’ (Table 2), were combined to form one determinant (e.g., ‘gender of previous children’ (Fig. 1)). A single code could also produce several determinants if multiple distinctive connections to perinatal mental health were identified from interviewee insights (e.g., ‘alcohol use’ code produced both ‘alcohol use by husband’ and ‘alcohol use during pregnancy’ determinants). As social determinants do not act in isolation, AW and NV also looked for relationships between the identified determinants to gain a fuller contextual understanding of perinatal mental health inequities and assess for potential compounding effects. These analyses yielded several themes regarding key social determinant networks, their mechanisms, and how they shape women’s experiences with perinatal CMDs. AW led the coding application and analysis processes, with any uncertainties discussed regularly with NV. Indian co-authors were also consulted frequently throughout these processes to ensure consistency between the data presented and the findings.

### Rigor and trustworthiness

This study was conducted and reported in adherence with the Consolidated Criteria for Reporting Qualitative Research guidelines for the reporting of qualitative research studies [68]. The page numbers in the manuscript where each item appears are reported in Table 3 below.

**Table 3:** Consolidated criteria for reporting qualitative research checklist. As developed by Tong, et al. (2007)

Topic	Item no.	Guide questions/description	Reported on page no.
**Domain 1: Research team and reflexivity**			
*Personal characteristics*			
Interviewer/facilitator	1	Which author(s) conducted the interviews or focus groups?	Pgs. 7–8
Credentials	2	What were the researchers’ credentials? (E.g., PhD, MD)	N/A
Occupation	3	What was their occupation at the time of the study?	N/A
Gender	4	Was the researcher(s) male or female?	Pgs. 7–8, 15
Experience and training	5	What experience or training did the researcher have?	Pgs. 8, 15
*Relationship with participants*			
Relationship established	6	Was a relationship established prior to study commencement?	Pg. 6
Participant knowledge of the interviewer	7	What did the participants know about the researcher? E.g., personal goals, reasons for doing the research	Pgs. 8, 16
Interviewer characteristics	8	What characteristics were reported about the interviewer/facilitator? E.g., Bias, assumptions, reasons and interests in the research topic	Pgs. 8, 15–16
**Domain 2: Study design**			
*Theoretical framework*			
Methodological orientation and theory	9	What methodological orientation was stated to underpin the study? E.g., grounded theory, discourse analysis, ethnography, phenomenology, content analysis	Pg. 9
*Participant selection*			
Sampling	10	How were participants selected? E.g., purposive, convenience, consecutive, snowball	Pgs. 6–7
Method of approach	11	How were participants approached? E.g., face to face, telephone, mail, email	Pg. 6–7
Sample size	12	How many participants were in the study?	Pg. 7
Non=participation	13	How may people refused to participate or dropped out? Reasons?	N/A
*Setting*			
Setting of data collection	14	Where was the data collected? E.g., home, clinic, workplace	Pgs. 7–8
Presence of non-participants	15	Was anyone present besides the participants and researchers?	Pgs. 7–8
Description of sample	16	What are the important characteristics of the sample?	Pg. 7
*Data collection*			
Interview guide	17	Were questions, prompts, guides provided by the authors? Was it pilot tested?	Pg. 8, Additional file 1
Repeat interviews	18	Were repeat interviews carried out? If yes, how many?	N/A
Audio/visual recording	19	Did the research use audio or visual recording to collect the data?	Pg. 8
Field notes	20	Were field notes made during and/or after the interview or focus groups?	Pg. 8
Duration	21	What was the duration of the interviews or focus groups?	Pgs. 7–8
Data saturation	22	Was data saturation discussed?	Pg. 7
Transcripts returned	23	Were transcripts returned to participants for comment and/or correction?	N/A
**Domain 3: Analysis and findings**			
*Data analysis*			
Number of data coders	24	How many data coders coded the data?	Pgs. 9–10
Description of the coding tree	25	Did authors provide a description of the coding tree?	Pg. 10
Deviation of themes	26	Were themes identified in advance or derived from the data?	Pg. 11
Software	27	What software, if applicable, was used to manage the data?	Pg. 10
Participant checking	28	Did participants provide feedback on the findings?	N/A
*Reporting*			
Quotations presented	29	Were participant quotations presented to illustrate the themes/findings? Was each quotation identified?	Pgs. 18–29, Additional file 2
Data and findings consistent	30	Was there consistency between the data presented and the findings?	Pg. 11
Clarity of major themes	31	Were major themes clearly presented in the findings?	Pgs. 18–29
Clarity of minor themes	32	Is there a description of diverse cases or discussion of minor themes?	Pgs. 18–29

Strategies that were employed to enhance credibility, dependability, confirmability, and transferability of the study findings are also outlined in Table 4 below.

**Table 4:** Measures taken to enhance credibility, dependability, confirmability, and transferability of study findings

Trustworthiness criteria	Method of incorporation
Credibility	Data triangulation: complementary focus group & individual interview formats used to collect perspectives from a range of stakeholder groups
	Member checking: interviewers frequently repeated, paraphrased, and clarified participants’ responses during interview sessions to ensure accurate comprehension [69]
	Reflexivity: the research team engaged in frequent reflexivity exercises during the analysis process to maintain objectivity and minimize potential for distortions in findings
Dependability	Initial triple-coder strategy: the first, second, and last authors worked independently to read through the transcripts and develop initial codes. These three sets of initial codes were compiled, compared, and refined to formulate the final codebook
	Peer debriefing: the first author engaged in frequent, rigorous debriefings with the last author during the codebook application process, which was applied using a single-coder approach due to resource and time constraints
Confirmability	Audit trail: records of each step of the research project were maintained
Transferability	Thick description: contextual information about the study setting, sample size, strategy, and characteristics, inclusion and exclusion criteria, and interview procedure and topics [see Additional file 1] are outlined in this report

### Authors’ positionality

The authors recognize that researchers’ attitudes, lived experiences, and backgrounds play an integral role in shaping the observations and meanings made from qualitative research [70]. Thus, before presenting our findings, it is helpful to understand the positionalities in which our research perspectives are grounded. The first author is a second-generation East Asian immigrant to the United States with 6 years of qualitative research experience. The last author is a female European scholar with a background in psychology and political science and a decade of research experience in global mental health in LMICs. The first author’s familiarity with both Eastern/Western and individualistic/collectivistic cultures from her immigrant background and the last author’s expertise and knowledge of Telangana from prior fieldwork helped to promote sensitivity and reduce interpretive bias throughout the research process. However, as both the first and last authors were raised in Western countries and do not identify as Indian, other authors that were born and raised in India and deeply familiar with the Telangana context were regularly consulted to ensure that the findings and interpretation were representative of the study setting. In particular, the interview content guides [see Additional file 1] were designed in collaboration with several colleagues from the George Institute for Global Health in India, including the third (SRT) and fourth (JKL) authors that led the FGDs with CHWs, caregivers, and women at risk for perinatal CMDs. SRT is a male researcher from India with a background in public health and extensive experience in qualitative and community-based research in rural areas. While conducting FGDs, his background as both a local and a husband to a woman from Telangana helped him to connect with participants. JKL is a female South Indian scholar with a background in medicine and women’s health and seventeen years of experience as a public health researcher and educator in Telangana. Her knowledge of the culture and health systems in the study sites and proficiency in Telegu also aided in rapport building during FGDs. Both SRT and JKL’s independent researcher roles were also repeatedly emphasized during group sessions to minimize the potential for social desirability bias that might arise from their insider statuses.

## Results

### Social determinants

Thirty-six social determinants of perinatal mental health were identified and mapped onto the six nested levels in Michaels, et al. [66] Mental Health and Well-being Ecological Model to form the novel socio-ecological model of perinatal mental health (Fig. 1). The innermost level contains socio-demographic factors while each subsequent outer level represents factors that are rooted in the women’s immediate support systems, healthcare institutions, community, government, and broader societal context.

Across the six levels of the socio-ecological model of perinatal mental health (Fig. 1), five distinct, yet interconnected social determinant networks were identified. The mechanisms and impacts of each network are discussed through the following five themes: attitudes about pregnancy outcomes, gender inequity, relationships & support, mental health stigma, and mental healthcare access (Table 5) [see Additional file 2 for definitions and illustrative quotations].

**Table 5:** Distribution of themes across the six levels of the socio-ecological model of perinatal mental health

Level	Theme 1: Attitudes about pregnancy outcome	Theme 2: Gender inequity	Theme 3: Relationships & support	Theme 4: Mental health stigma	Theme 5: Mental healthcare access
Society	X	X	X	X	X
Policy		X		X	X
Community		X	X	X	
Organizations				X	X
Interpersonal relationships	X	X	X		
Individual	X	X	X		

#### Theme 1: Attitudes about pregnancy outcomes

Attitudes about pregnancy outcomes were discussed in relation to social determinants across the societal (e.g., son preference/daughter aversion, discrimination & exclusion), interpersonal (e.g., infant gender outcome, gender of previous children), and individual (e.g., history of pregnancy loss, number of previous pregnancies) levels of the socio-ecological model of perinatal mental health (Fig. 1).

##### Gender preference

There was general agreement across all participant groups that son preference was a leading concern for women during the perinatal period. Participants indicated that pregnant women were often subject to strong internal and external pressures to produce at least one male heir for their spouse’s family. Thus, women’s mental health during the perinatal period frequently depended on the gender outcomes from their previous pregnancies, with those that had multiple daughters experiencing significant pressure to produce a son. But regardless of whether they already had sons or not, *“raising a daughter is considered a burden… because*,* you know*,* for education and then getting her married” (Psychiatrist*,* IDI8).* Despite this, a few women expressed a desire to have daughters, with other participants discussing several cases wherein women with multiple sons faced external pressure to produce a daughter. Participants reported that these expectations regarding infant gender could cause severe distress and lead women to pursue abortions, regardless of the desired gender.

In the aftermath of undesirable gender outcomes, women were susceptible to abuse and neglect from their husbands and in-laws. In extreme cases, participants noted that this has led to suicide and infanticide attempts.

##### Health outcomes

Many antenatal women, especially those with co-occurring gestational health issues, reported fears of adverse maternal and infant health outcomes like birth defects, prematurity, and pregnancy loss and complications. Equally, these events caused women immense grief and distress in their aftermath. Adverse outcomes from previous pregnancies were also seen as a high-risk factor for poor mental health during subsequent pregnancies, as women feared repetition of these negative outcomes. Many women, especially primigravids, also worried about the delivery process, with a preference for natural over cesarean deliveries.

Despite beliefs that divine intervention contributed towards determining conception, health, and infant gender outcomes, participants agreed that the blame for pregnancy losses and poor infant health (e.g., prematurity, low birth weight, infections) was placed on the women. Many cited the link between fetal and maternal health, both mental and physical, during pregnancy in the rationale behind this stigma:

“They say she has killed her baby… she didn’t take proper food, [or] take good care… They [find] many reasons to blame her” (Caregiver, FGD6)

In addition, participants highlighted practices of social exclusion and seclusion in the immediate aftermath of pregnancy losses, undesirable gender outcomes, or cesarean section deliveries that served to prevent ‘transmission’ of the negative outcome to other pregnant women in the village. This practice further directed blame for these outcomes towards women and barred access to mental health support during a time of mourning, causing greater emotional distress.

#### Theme 2: Gender inequity

Discussions of gender inequity occurred across the societal (e.g., women’s roles & responsibilities, attitudes about domestic violence), policy (e.g., nutritional services), community (e.g., nutritional access, employment conditions & nature), interpersonal (e.g., alcohol use by husband, involvement in pregnancy planning), and individual (e.g., maternal health conditions) levels of the socio-ecological model of perinatal mental health (Fig. 1).

##### Women’s limited agency and autonomy

Participants reported that in-laws and husbands held the power to make reproductive decisions for women, forcing some to undertake or retain pregnancies against their will and many to carry pregnancies early and frequently during marriages. This often affected women’s physical health and further diminished their agency and autonomy:

“If back-to-back deliveries keep happening, then we will become weak. So [the women] will be totally dependent on them (the family)” (Self-help group leader, IDI5)

Such external pressures to undertake and complete unwanted pregnancies were not only immensely distressful, but could also impair women’s attachment to their children and perpetuate poor perinatal mental health across generations:

“One woman who had clinical depression in [her] first pregnancy… she was born to a mother who never wanted to have children [and she] did not have a relationship [with her] mother. [When she had her] first child, which happened because she was married, she did not really want the child… She connected with her mother not wanting to give birth” (Mental health professional, IDI2)

##### Prioritization of family

Participants reported that women were expected to serve their families through food preparation, housework, and childcare, and prioritize their families’ needs above their own:

“[The] concept of even… thinking your own thoughts [and] feelings… is not going to work… The minute you say ‘I’… you’re out of the family” (Clinician, IDI1)

Pregnancy and the time after birth represent a unique loss of individualization as the mother gains responsibility for nurturing the child. The woman’s role as the carrier and carer of the baby was seen to contribute to the short-term prioritization of maternal mental health by husbands and in-laws during the perinatal period:

“They will care for the sake of the baby… later they don’t care… Baby is very important. Wife is for use only” (Women at risk for perinatal CMDs, FGD9)

Together with the negative consequences inflicted for undesirable pregnancy outcomes, this sentiment suggested that women’s bodies were valued only as reproductive vessels for their family of marriage.

##### Conflicting responsibilities during pregnancy

Women’s responsibilities to protect both their family’s livelihood and lineage frequently came into conflict during the perinatal period, especially for those of lower socio-economic status. Notably, participants indicated that pregnant women were often expected to continue doing housework despite physical limitations and the risk of repercussions on their health. Navigating these conflicting demands could evoke mental anguish. Poor economic well-being augmented this issue, as many pregnant women were also expected to continue their employment in the tobacco or agricultural industries to support their family’s livelihood. In addition to the strain caused by navigating conflicting demands, these women also experienced distress from difficulties in balancing housework and job responsibilities. This was further compounded by high alcohol consumption among men, as the *“kids’ responsibilities are only given to single person [i.e. the woman*,* and] we suffer to bring the groceries home” (Caregivers*,* FGD6).*

Pregnant women were also expected to prioritize their family’s nutrition above their own. Participants reported that some women were only be allowed to eat after serving their families or were prevented from accessing Anganwadi nutritional services. Women often struggled to fulfill these familial expectations, which limited their nutritional intake and quality, while sufficiently supporting fetal and maternal health. Maternal malnutrition was further exacerbated by poverty and food insecurity.

##### Domestic violence

Participants reported domestic abuse and violence towards perinatal women to be very common. Both women and healthcare providers indicated that women were at increased risk for domestic violence if their reproductive or household duties were perceived to be inadequately fulfilled, or if their partner frequently consumed alcohol. This presented an additional burden during pregnancy and risk factor for mental health:

“We get upset because the extra burden of carrying the baby and being with an abusive husband is so much stress. We think that it would have been better if we were unmarried rather than suffering like this” (Caregivers, FGD6)

However, peers often encouraged women “*to try to maintain the toxic relationship [as] they feel breaking a relationship is more traumatic than undergoing [domestic violence]*” (Psychiatrist, IDI8). Help seeking was also limited because:

“Many people think it’s only… life-threatening physical abuse… whereas simple slaps or just hitting or punching is also domestic violence… Physical or verbal abuse is very rampant but many women… have accepted [it] as a part of life” (Psychiatrist, IDI8)

#### Theme 3: Relationships and support

The significance of women’s relationships and support systems was also noted across the societal (e.g., cultural & faith-based practices), community (e.g., community relationships, financial well-being), interpersonal (e.g., peer relationships & support, relationship & support from in-laws, husband, and own family), and individual (e.g., alcohol use during pregnancy) levels of the socio-ecological model of perinatal mental health (Fig. 1).

Helping with household chores, providing reassurance, and gifting food were common forms of support provided to perinatal women by family and community members. Participants indicated that the gifting and consumption of alcoholic palm wine, or ‘toddy’, during pregnancy was still practiced in some villages due to beliefs that the practice supports fetal development, maternal health, and childbirth.

##### Relationships with husband and in-laws

Due to the extended family structure that is common in India, support from in-laws was found to significant impact women’s perinatal mental health. This support was variable; some mothers-in-law were reported to try to come between couples and cause distress while others were willing to oppose their own sons to support their daughters-in-law’s mental health. Husbands also played an imperative role in protecting perinatal mental health:

“If the husband says [no matter] a boy or girl, we will take care, then wife will not feel bad. But if he goes on insisting on a boy baby, then she will become anxious” (Caregiver, FGD7)

However, participants noted that husbands’ support could be impaired by alcohol use and the influence of the woman’s in-laws. When husbands were uncooperative, intervention from the woman’s parents and ASHAs could increase support.

##### Reliance on the family of origin

Pregnant women commonly migrated to their family of origin near the time of delivery in their first pregnancy. This also occurred in subsequent pregnancies if in-laws were not providing sufficient care: *“When she is not having a proper diet… we counsel her mother-in-law to send her to her parents’ house” (Gynecologist*,* IDI4).*

The woman’s family of origin was usually supportive and protective against perinatal CMDs. Women’s parents expressed willingness to pay for expensive private antenatal care over free governmental services and seek mental healthcare for their daughters, if necessary, despite stigmas associated with doing so. This may stem from reduced concerns about infant gender, a stronger sense of kinship, and greater prioritization of maternal health by her family of origin.

Additionally, the family of origin was responsible for funding dowry payments, baby showers, and antenatal care. The financial strain of these payments was reported to cause women distress during the perinatal period:

“[Getting] pregnant within one year [of marriage] will be problematic for [her parents’] expenditures [especially when there is] pressure from mother-in-law [for private institutional deliveries]” (ASHA, FGD5)

Conversely, the inability to pay for antenatal care could also be a source of tension during the perinatal period.

##### Community relationships

Women were seen to seek community support when their families were unsupportive or causing them mental distress. While support from close friends was typically seen as protective, other community members “*may not listen… properly or they may gossip [and] mock at us*,* they never try to solve the issues. They just add fire into the problems” (Caregiver*,* FGD6).* Many also indicated that women might not seek help from community members because of expectations to keep family matters private and fears of negative consequences from in-laws for discussing these matters with others.

#### Theme 4: Mental health stigma

Mental health stigma was discussed across the societal (e.g., mental health stigma), community (e.g., community relationships, awareness of perinatal mental disorders & services), policy (e.g., Sakhi centers), and organizational (e.g., perinatal mental health service accessibility) levels of the socio-ecological model of perinatal mental health (Fig. 1).

##### Societal views reinforce stigma

Participants noted that the term ‘mental illness’ was typically conceptualized as extreme psychosis, with limited awareness of the variability in condition type and severity. Women affected by poor mental health were generally perceived to be ‘careless’, but pregnancy added connotations of selfishness and weakness due to the emphasis on their familial responsibilities during this period. In addition, caregivers and healthcare providers noted that perinatal CMDs were typically associated with and recognized through drastic somatic and behavioral indicators including poor breastfeeding habits, unsociable behavior, and physical weakness, which perpetuated stigmatization and hinder help-seeking.

##### Stigma inhibits access to care

The normalization of anxious and depressive feelings during pregnancy and the fear of familial and social consequences was reported to prevent perinatal women from seeking care for mental health challenges. Participants disclosed that such concerns could also be met with responses from CHWs like ASHAs, ANMs, and AWWs that reinforce stigmas and discourage further help-seeking behavior. Women may have also abstained from pursuing treatment even if they were referred for care because *“walk[ing] into a mental health facility [or] Sakhi center is labeled as having a very big problem whereas women [go] for smaller problems also” (Psychiatrist*,* IDI8).*

#### Theme 5: Mental healthcare access

Barriers to mental healthcare were discussed in connection to social determinants across the societal (e.g., traditional healers & remedies), policy (e.g., Sakhi centers, inclusion of mental health in routine antenatal care), and organizational (e.g., provider knowledge of perinatal mental health conditions & services, perinatal mental health service availability) levels of the socio-ecological model of perinatal mental health (Fig. 1).

##### Community health workers are the bottleneck

Our results indicated that ASHAs, AWWs, and ANMs were the first contact point for perinatal women in the community and were therefore frequently tasked with recognizing and providing informal support for perinatal CMDs at the local level:

“It’s we. We are everything in the sub-center” (ASHA, FGD4)

“You are the doctors; you are the nurses” (Interviewer)

Despite this, participants noted that CHWs were not formally trained to provide mental health support or screening and had to rely on the women or their families to disclose signs of perinatal CMDs and request initiation of treatment and referral processes. However, the lack of privacy during ASHA’s routine home visits and stigmas discouraged women from disclosing their concerns to CHWs. The limited awareness of referral routes that these providers had could prohibit access to specialized care even when women came forward.

##### Challenges in referral pathways

In addition to CHWs, participants described two other routes through which pregnant women received referrals for psychiatric care: Sakhi centers and gynecologists. Sakhi centers are government-administered community domestic violence resource centers whose utilization, as illustrated above, was hindered by women’s fears of being labelled as mentally ill or as having familial issues.

As mental health screenings had not been implemented into routine antenatal care, gynecologists had to rely on their own perceptions to identify perinatal CMDs for psychiatric referrals even though they were not trained to do so. Thus, the timing of referrals and effectiveness of treatment was practitioner dependent.

##### Lack of mental health specialists

Even when women were referred for treatment, healthcare workers noted that specialist care was frequently unavailable. Since psychiatrists were only present at district hospitals, access to these providers was restricted by geographical distance and limited mental health resources at facilities. This increased the burden of mental health support on CHWs and prevented women from receiving proper care for perinatal CMDs:

“Where do these pregnant women with mental health problems get help? Will they get any help?” (Interviewer)

“They don’t get any help” (ASHA, FGD5)

Instead, alternative traditional options for care and treatment of perinatal CMDs were commonly sought, including prayers at dargahs, thread-tying, and other rituals.

### Interventions targeting social determinants

**Fig. 2:**
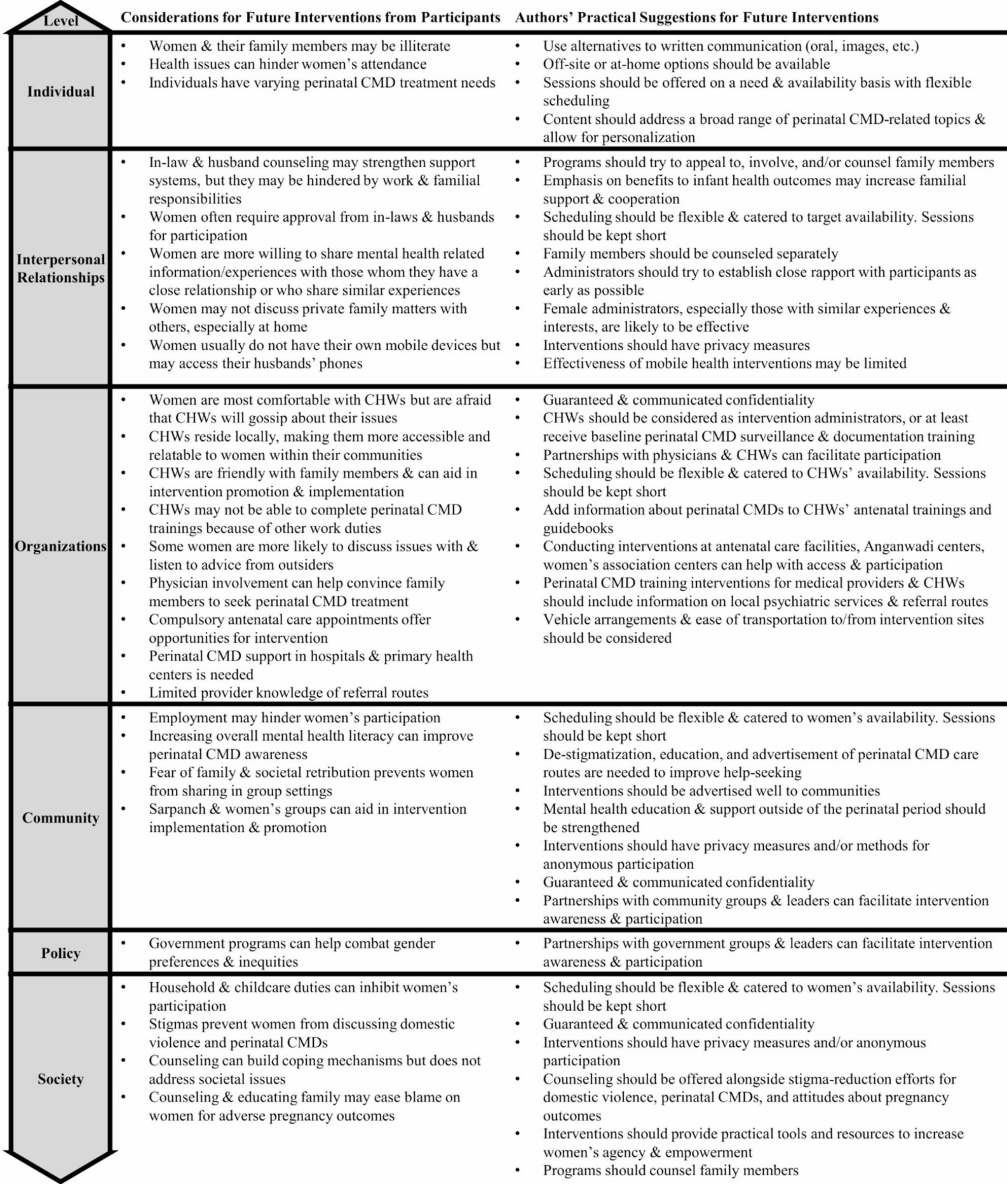
Considerations, potential challenges, and auggestions for future intervention design and implementation, as applied to the socio-ecological model of perinatal mental health. Adapted from Devooght, et al. (2023) [71] and Michaels, et al. (2022) [66]. CHW = community health worker, CMD = common mental disorder

Considerations for future interventions targeting social determinants of perinatal CMDs from participants and corresponding practical suggestions from the researchers to address them are detailed in Fig. 2. These insights were categorized based on their level of influence within the socio-ecological model of perinatal mental health [66].

## Discussion

### Social determinants

This qualitative study sought to understand what and how social determinants affect perinatal CMDs in rural villages in Telangana, a state in South India. Drawing from interviews with women at risk for perinatal CMDs, their caregivers, and other mental health stakeholders, a total of 36 social determinants acting at individual, interpersonal, organizational, community, policy, and societal levels (Fig. 1) were identified. Relationships between these determinants and their context, concepts, and impact on perinatal mental health were explored through five themes.

First, child gender preference was discussed as a major risk factor for perinatal CMDs. The negative relationship between son preference and perinatal mental health outcomes has been well-documented across India [72] and other South Asian countries including Nepal [73, 74], Pakistan [75, 76], and Bangladesh [77, 78]. In line with such observations, our findings indicate that despite growing daughter acceptance in South India [79], preference for male children continues to strongly influence perinatal mental health outcomes in the region [13, 80–83]. This preference has been attributed to patrilineal familial inheritance patterns [84], Hindu funeral customs [85], and improvements to women’s status and decision-making power from bearing male children across India [86, 87]. However, our results show that sons are not just preferred, but daughters are actively disfavored. This ‘daughter aversion’ phenomenon has been previously discussed in connection with dowry payment customs across Northwest [86] and South India [79, 88, 89], but our results indicate that baby shower and delivery costs associated with women’s families of origin also contribute. Our study is also the first, to our knowledge, to link worries about these costs, which occur in quick succession and close to the perinatal period, with perinatal CMDs in an Indian or South Asian context. Women’s concerns about the financial burden that their own daughters will pose, which prominently contributes to female infanticide in India [89–91], and fears of subjecting them to gender-based discrimination and inequities [92] should be investigated in relation to perinatal CMDs in India, especially since the latter has been recognized to shape women’s preferences for male children in similar South Asian contexts like Pakistan [93, 94].

Beyond the pressures to bear male children during pregnancy, our findings agree with previous observations across South and Central India that postnatal women are susceptible to hostility, blame, and maltreatment for birthing children of undesirable genders [51, 82, 95]. Similar disruptions to women’s relationships and support provision have also been noted to occur in the aftermath of induced and spontaneous abortions, stillbirths, and other adverse pregnancy outcomes in India [96–98]. The fear of repetition and worsening of these social consequences, including becoming labelled as defective [99, 100] or ‘taken out of the family’ [95], likely contributes to the heightened stress that we observed amongst women with multiple daughters or previous pregnancy losses in Telangana. Previous research from South and Central India has also pointed to pronatalist attitudes that emphasize motherhood as a measure of womanhood and misconceptions about causes of pregnancy losses as contributors towards women’s self-blame, grief, and reluctance to discuss and seek care for such experiences [97, 98, 100]. The seclusion and exclusion of women that have suffered pregnancy losses in India [95, 101] can further exacerbate their distress and prevent access to support systems and others with similar lived experiences, impeding their ability to cope with grief. Similar findings have also been reported in South Asian LMICs like Bangladesh [102], Nepal [103], and Pakistan [104], wherein beliefs that attribute pregnancy losses to possession by evil spirits also contribute to the isolation and shunning of affected women. As elevated cortisol levels associated with poor mental health can disrupt ovulation and impair fetal growth [105–107], the prolonged, severe distress that these social consequences inflict upon women may also perpetuate cycles of infertility, adverse pregnancy outcomes, and perinatal CMDs. Thus, there is an urgent need for interventions that stress women’s worth regardless of motherhood, comprehensive education on infertility and pregnancy complications, and bereavement care for women that have experienced adverse pregnancy outcomes to break these cycles [108–110].

Additional ramifications of gender inequities on perinatal CMDs were discussed in the third theme. Our results indicate that women’s limited control over reproductive decisions in India, including antenatal service usage [111], family planning [112], and mode of delivery [113], negatively affects their perinatal mental health. Previous explorations of women’s disempowerment across Pakistan [94] and India [113, 114] have attributed this to the patriarchal and patrilocal structures that grant decision-making power to the women’s husbands and in-laws and place them at the bottom of their household hierarchies. A qualitative study in Pakistan also demonstrated that women may be especially hesitant to challenge reproductive decisions made by their in-laws and husbands during the perinatal period, as this could lead them to lose crucial support and exacerbate their distress [94]. The collectivist nature of Indian society may also compel women to comply with such decisions for the benefit of their families [115], which can prevent help-seeking for perinatal CMDs and perpetuate perceptions of affected women as selfish.

The impact of navigating conflicting gender roles on women’s mental health in India has been described [116, 117] but, to our knowledge, has not been comprehensively investigated in the perinatal context prior to this study. Our results show that perinatal women are simultaneously pressured to prioritize their family’s livelihood and nutrition and protect maternal and fetal health. Both attempting and failing to balance these conflicting responsibilities can cause distress, especially as women often lack the power to make decisions regarding the food that they eat and their household duties while living with their husbands and in-laws [95]. Expectations for women to eat only after their families have finished, which were observed among a quarter of households in the 2011 India Human Development Survey [118, 119] and across South Asia [120, 121], have been associated with undernutrition and poor mental health even before pregnancy [122]. Our study shows that such gendered food discrimination and its effects on undernutrition can extend into the perinatal period, thus compounding women’s risk for perinatal CMDs, low infant birth weight, and infant mortality [119, 123]. Despite this, as Roberts, et al. (2012) also noted, these women are shamed for not eating healthily and taking appropriate rest in the event of these adverse outcomes.

Women’s ability to navigate these gender roles is worsened by financial and food insecurity. Our findings show that these conditions can drive perinatal women to hold external employment that can worsen physical strain and familial responsibilities. Economic stress has also been cited as a source of domestic violence in South India [91], and women’s limited empowerment and dependence on their families, especially during and after pregnancy, increases their vulnerability to such violence [124, 125]. Food insecurity can also compound the effects of maternal deprioritization on their malnutrition. While governmental AWW services have sought to alleviate maternal malnutrition through low-barrier nutritional support across India [126], our results indicate that collectivist and patriarchal norms that devalue women’s nutritional priorities in their families can hinder them from utilizing these services. These findings illustrate the need for multi-faceted approaches to perinatal mental health that challenge gender norms, strengthen familial support, advocate for maternity leave policies, and deliver financial and nutritional support.

Despite societal expectations for perinatal women to serve their families of marriage, our findings show that the support that they receive in return often varies. This is likely because a woman’s status within her family of marriage in India depends upon her fertility, ability to produce at least one son, and age in relation to her sisters-in-law [95, 115, 127]. Consistent with Kazi, et al. (2006)’s insights from Pakistan, our results posit the relative strength of the husband’s relationships with the woman and his mother to be an additional determinant of perinatal support. This competition may be attributed to the concentration of decision-making power in the patriarchs within South Asian families. In agreement with evidence from Pakistan [128], Bangladesh [129], and Nepal [130], our study demonstrates the nature of these relationships to be strongly associated with perinatal mental health outcomes; negative relations between women and their husbands or in-laws, characterized by physical or verbal abuse [129] and excessive in-law involvement in household decisions [128], increase women’s risk for perinatal CMDs. Conversely, positive relationships can provide emotional support, facilitate access to antenatal care, and even reduce women’s burden of household responsibilities during the perinatal period [128]. As the nature of these relationships play a substantial part in determining women’s risk for perinatal CMDs and ability to access care across South Asian LMICs, initiatives to strengthen support from husbands and in-laws are needed.

Although women are incorporated into the husband’s family after marriage, their family of origin remains a tremendous source of financial and emotional support in India. Generally, women with close ties to their parents and brothers are more likely to realize their own needs and be treated better by their in-laws throughout their marriages [127]. Our findings suggest that such support can allow women to recognize and seek care for perinatal CMDs, improve the quality of support from the in-laws, and reduce harm to women after pregnancy losses.

In line with previous studies across Bangladesh [131], India [49, 51–53, 56], and Pakistan [132], we identified stigma as a key barrier to seeking and receiving perinatal mental health treatment. For instance, a majority of surveyed family members of postpartum mothers in Bangalore expressed beliefs that women with postpartum depression cannot make any decisions (90.1%), be good mothers (70.8%), or take care of their own children (74.2%) [51]. Our findings posit the attribution of mental illnesses to character deficiencies and the association of the term ‘mental illness’ with extreme conditions as fundamental ideas underpinning these attitudes. Societal expectations of motherhood in India [133] also contribute to these characterizations of women with perinatal CMDs as ‘bad mothers’ [51]. Fears of becoming labeled as a bad mother may exacerbate the effects of mental health stigma on women’s help-seeking [134] and treatment adherence [135] during the perinatal period [136]. Repercussions that these labels have on their family’s reputation can also hinder women from seeking care. The internalization of these negative connotations, which is stronger in collectivistic cultures, impacts women’s self-esteem, self-efficacy and feelings of isolation [137–139]. Thus, both perinatal CMD literacy and support strategies are needed to address public stigmas and empower affected individuals [140, 141].

Inaccessibility of perinatal mental health services poses another barrier to care. Notably, psychiatric services are primarily offered at tertiary care centers in India, whose accessibility to women residing in rural villages is often barred by transportation difficulties, geographical distance, and high costs [142–144]. Instead, these women receive most of their perinatal care from primary health centers, wherein trained mental health professionals are largely absent, and CHWs that are not trained to recognize and address perinatal CMDs and may even carry stigmatizing attitudes towards affected women [145]. Thus, women’s access to perinatal CMD care in rural South India is dependent on their ability to overcome stigmas and initiate care. As CHWs often develop close relationships with perinatal women and gain comprehensive understandings of their lives through home visits [146], they should be trained to recognize perinatal CMDs, provide basic counseling, refer affected women for psychiatric treatment, and address mental health stigmas. The success of ASHA-led mental health interventions in South India [147] and their established role in reducing maternal and neonatal mortality further illustrate their potential to address unmet perinatal CMD needs [148]. Several CHW-mediated perinatal mental health programs have already demonstrated success in similar South Asian LMICs. This includes the ‘Thinking Healthy Programme’ in Pakistan, which significantly reduced the prevalence and severity of perinatal depression *via* CHW-delivered cognitive behavioral therapy sessions [149]. The intervention’s robust impact on perinatal depression was sustained among recipients even seven-years after initial administration [150], making it an attractive model for perinatal mental health interventions in India and other South Asian LMICs with similar socio-cultural contexts.

Our results demonstrate that integrating perinatal mental health screening and care into routine maternal, newborn, and child health services is needed to improve perinatal CMD outcomes in Telangana. Integrated maternal mental healthcare models in other Indian states like Kerala [151] and Karnataka [152] are promising and warrant replication. However, national policy solutions are urgently needed to expand the necessary personnel, infrastructure, and funding to address perinatal CMDs at a population level [148, 153]. This may be done by incorporating perinatal mental health into the objectives of the National Mental Health, Pradhan Mantri Surakshit Matritva Abhiyan, and Reproductive, Maternal, Newborn, Child, and Adolescent Health Programs [154] and scope of the 2014 National Mental Health Policy. To support provision of such integrated services in India and address the burden of perinatal CMDs across other South Asian LMICs, further in-depth research on policy needs and best solutions in these contexts should be urgently pursued. Manolova, et al. [155] eight pillars for the integration of perinatal mental health into maternal and childcare in LMICs (screening, individualized support, psychoeducation, psychosocial support, special programs for unique populations, training/technologies, high intensity treatments and referral programs, and supervision of healthcare workers) should be considered to ensure the success of these future programs.

### Interventions targeting social determinants

Considerations, potential challenges, and suggestions for future perinatal mental health interventions targeting these social determinants were also mapped onto the socio-ecological model of perinatal mental health (Fig. 2). Our findings indicate that perinatal women, their families, providers, and community members are all suitable intervention targets. To prevent and address harm from perinatal CMDs, perinatal women will benefit from counseling, support programs [156], and other strategies that empower them to practice self-care, pay attention to their mental health, and seek professional help when necessary [157]. As husbands and in-laws influence women’s perinatal CMD outcomes, care attainment, and intervention participation, programs should also aim to educate and strengthen support from these family members. Emphasizing the benefits of protecting perinatal mental health on infant outcomes can garner familial support but should be supplemented with material that stresses women’s worth regardless of motherhood. Physician endorsement of and involvement in interventions may also boost their acceptance, encourage families and communities to prioritize maternal mental health, and optimize referral efficiency [95, 158, 159]. Along with CHWs, antenatal physicians should also be targeted for training interventions to improve perinatal CMD recognition, documentation, and referral. Lastly, improving community mental health literacy can help reduce stigma. Lectures, case scenarios, role-plays, and contact-based education are promising methods for such initiatives [160].

Accessibility is a key consideration for intervention design and implementation. Due to women’s household responsibilities, possible health restrictions, and limited autonomy, scheduling and attendance policies for initiatives that target this population should be flexible and cater to their availability [161], with appropriate off-site or at-home alternatives. Low literacy rates [162], poor mobile device access, and lack of privacy at women’s homes can limit these options. Alternatively, mentor and peer pairings can enable participants to review missed content while also fostering trusting relationships. Interventions may also benefit from using pictorial illustrations and local intervention administrators to ensure that materials are presented in an approachable, comprehensive manner [163]. Further, providing transportation to program sites [161], holding interventions at antenatal care facilities, and keeping sessions concise while providing ample time for sharing, reflecting, and meaningful learning may also ensure accessibility.

Individual and group formats both have their strengths. Logistically, group interventions are time, space, staff, and financially efficient [164]. Although stigmas regarding perinatal CMDs and their treatment may affect women’s willingness to attend and discuss personal issues in support groups, support and care from others with shared experiences can improve perinatal CMD outcomes [139]. Thus, strategies to build rapport with intervention administrators (i.e. appointing women with experience of perinatal CMDs and CHWs), establish trust between participants, and protect privacy and confidentiality are imperative for group interventions. One-on-one perinatal CMD interventions can provide more individualized care but may also be hindered by and perpetuate stigmas related to attending care. To address these stigmas and the need for familial approval, interventions may benefit from incorporating other perinatal health topics that are not explicitly related to perinatal CMDs like nutritional recommendations, hygiene, and child development into their curricula.

### Limitations

While this study provides valuable insights on social determinants of perinatal mental health in rural Telangana and interventions to address them, it is not without limitations. The first comes from the limited number of participants and great breadth in their roles, which limit the generalization of our findings. Participants may have also had a particular interest in the topic, ability to attend the sessions (i.e. access to childcare, approval from husbands and their families, fewer housework or employment responsibilities), and willingness to discuss sensitive topics and experiences that may not be representative of the general population or women facing further marginalization, including adolescent mothers who were excluded from the study. Thus, supplementary work that focuses specifically on women with additional vulnerabilities (e.g., adolescent and single mothers, women with disabilities) and explores the impact of intersectionality on perinatal mental health in the region should be undertaken. Further, the selection for at-risk women by ASHAs and ANMs increased the potential for gatekeeper bias in our study, especially since we found that these local CHWs typically lack perinatal mental health expertise and training. While the recruitment process was monitored by researchers to minimize the impact of such biases, we acknowledge that this method likely caused women that could not report mental health struggles to CHWs, were not perceived as distressed, and did not experience challenges that ASHAs could recognize to be underrepresented in our sample. Similar work should employ validated screening tools like the Patient Health Questionnaire-9 and Generalized Anxiety Disorder-7 to ensure consistent, representative sampling of these participants.

We also acknowledge that though we were able to identify an expansive array of social determinants, our list is not exhaustive. For instance, information about income, caste, religion/spirituality, adolescent pregnancies, and education level was not collected from women at risk for perinatal CMDs, further restricting the generalizability of our findings and our ability to understand the effects of such factors on perinatal mental health. There was also limited representation of policy-based determinants among our data and findings, which may be due to a lack of existing perinatal CMD-related policies in Telangana [142], low awareness of existing programming among interviewees, and insufficient representation of policymakers among our study sample. Future work could employ mixed methods to explore these factors in depth and understand their influence on perinatal CMD experiences and outcomes. While the authors have completed a complementary review of existing perinatal CMD-related policies and programs in Telangana and Haryana [142], further inquiries across India and other South Asian LMICs should be undertaken to better understand and address gaps in psychosocial care.

Additionally, stigma, the presence of a male interviewer, and group interview formats may have affected participants’ responses and willingness to share in FGDs. Women may not have felt comfortable discussing their mental health struggles or family dynamics in the presence of peers, especially as many referenced rumors among community members as a stressor and barrier to perinatal mental healthcare. Previous research in Bihar, India [165] has also shown that women may not be permitted to or comfortable with disclosing information about stigmatized, female-specific health topics like domestic violence, contraception, and sexual behavior to male researchers. Despite the inclusion of local facilitators, the status of the researchers as ‘outsiders’ to the specific villages may have also deterred women from sharing insights that would reflect poorly on their local communities.

## Conclusions

This study identified 36 social determinants that impact perinatal mental health outcomes in rural Telangana, India. Stigmatization and the lack of well-trained, accessible providers and antenatal screening tools were found to be barriers to perinatal CMD treatment while financial and nutritional insecurity, conflicting gender roles, pressures to bear sons, and domestic violence contribute to negative perinatal mental health outcomes. As these factors often exert influence on one another within and across multiple levels of influence, intersectoral actions that support health through nutrition, gender equity, welfare, education, and healthcare reform are needed to effectively address the perinatal CMD burden in rural South India.

## Supplementary Information

Below is the link to the electronic supplementary material.

Supplementary Material 1

Supplementary Material 2

## Data Availability

The dataset analyzed in the current study is available from the corresponding author upon reasonable request.

## Abbreviations

ANMs: Accredited nurse midwives
ASHA: Accredited social health activist
AWW: Anganwadi worker
AYUSH: Ayurveda, Yoga & Naturopathy, Unani, Siddha, and Homeopathy
CHW: Community health worker
CMD: Common mental disorder
FGD: Focus group discussion
IDI: In-depth interview
PRAMH: PeRinAtal Mental Health
