# Supplementary material for: Perinatal mental health and its social determinants: qualitative findings from rural Telangana
Source: BMC Psychiatry. 2026 May 23;26:553. doi: 10.1186/s12888-026-08066-1 (PMC13390382; doi:10.1186/s12888-026-08066-1)
Supplement: Supplementary file 1 — Supplementary Material 1 [file 12888_2026_8066_MOESM1_ESM.docx]

# **Additional file 1: Interview topic guides**

**Additional file 1.1: Topic guide for focus group discussions with women at risk for perinatal common mental disorders**

***Overview***

This is the topic guide for interviews. The questions below are prompts to explore details on perinatal mental health in rural India. The questions should be seen as prompt for triggering discussions and conversations on topic areas, rather than attempting to cover all points listed.

***Introduction***

Thank you for taking part in the PRAMH study and this focus group discussion.

The wider research aim of the PRAMH study is to understand the context and state of perinatal mental health systems, services, and resources in India, specifically in the states of Haryana and Telangana.

To investigate this and understand the context and details of perinatal mental health in rural areas of Haryana and Telangana, we have invited you as experts to discuss relevant topics and help answer our questions.

The focus group discussion will explore a number of topics around perinatal mental health, for instance around people living with mental health problems, how they seek and receive help, cultural values and norms, barriers to get help, and stigma and discrimination.

The focus group discussion should take no longer than 1.5 hours. You are free to pause, stop, ask questions, and withdraw from our conversation at any time, without giving reason and without any consequences for yourself.

***List of the topic areas and examples of prompts/questions that could be explored:***

*Intro & context*

1. Can you tell us about your home and where you live and how your day looks like?

2. What work you do, how many children you have, married/widowed, etc.

*General mental health knowledge, attitudes/perception and behaviour*

3. Did you ever see anyone suffering from mental illness?

4. Some mental health problems are not as extreme, it may mean that people are feeling very low/depressed, or very anxious/worried. Have you met people with these problems?

- Mental illness may be understood as: Physical appearance: roaming on the road, dirty = severe MD. But we are talking about CMDs
- Perception that depression, anxiety is not the same mental disorders/ suicidal thoughts

5. How do you feel when you see someone suffering from their mental health?

- Would you work with them/ live with them? Be friends with them? Be married to them?

6. What are the reasons you think that people are having mental health problems?

- E.g. Because of illness, because of something in the past (previous birth), having a girl child, exhaustion, household responsibilities, family problems, etc.

7. How do people in your community/village take care of someone with a mental health problem?

8. Would you take care of someone if they had a mental health problem? Within your family (how close?)

*Women with mental health problems in pregnancy and after childbirth*

9. What are the mental health problems you know in pregnant women/ mothers?

10. Do you know how the pregnant women/mothers with mental health problems take care of themselves?

- Do they use kollu/alcohol to cope?

11. Where do pregnant women/mothers with mental health problems go to get help?

- What treatments/therapies are available? ASHA, family, spiritual healer, etc.?
- What help would the women want?

12. What challenges are there when she would like to get help for mental health?

13. Who is the best person the woman can go to for help? Or group?

14. Do you think the husband and family would support a woman if she needs help for mental health?

- And if this would help the baby?

15. How can the family/community help the women with mental health problems?

16. When women during pregnancy and after birth experience a lot of distress, they might think of taking their own life. Have you ever heard of an instance where a woman in such a situation who wanted to die?

- Do you feel it is OK to talk about suicidal thoughts/suicide during pregnancy and after birth?
- If a woman in pregnancy or after birth is thinking of taking her own life, what will family/community around her do? What do you feel they should do?

17. If a woman feels low/depressed or very anxious/worried, this can affect her physical health and that of her baby. Would this be a good reason to seek help for the negative feelings?

18. Some people with mental health problems drinking alcohol, e.g. the village drink (Kollu), a problem in your village?

- Drinking it often/ habitually?
- During pregnancy?

*Women’s own experiences*

19. When you are/were pregnant, who are the people who are supporting you? And after birth?

20. How did you feel/your mental health during your first-time pregnancy? And how was it different in the second pregnancy?

- What were the thoughts?
- How was it after the baby was born?
- During pregnancy or after birth, did you experience feeling low or anxious/tension?

21. Whom did you approach if you needed help?

22. You said you work in XYZ (see responses above to Q2). Were you interested to work during that time of emotional distress/feeling low/anxious?

- How was it to do the work?

23. During that time of emotional distress/feeling low/anxious, were you interested in meeting friends/ family?

- Did you tell them about how you were feeling?

24. How is it in your family, would your husband and his mother understand if you are feeling unwell or if you don’t work? Would you get food?

25. Are/were you staying with your mother before and after giving birth?

- How long did you stay?
- Was it different for first/subsequent pregnancies?

26. Before getting pregnant, was it the right time for you/when you wanted to get pregnant?

- Was it earlier or was it later than what you wanted?

27. When you had your baby, how was the health of the baby? What hopes did you have?

- Did you want to have a girl or a boy?
- Did your husband/in-laws want you to have a son?

28. After your child was born, did your family treat you differently

- Depending on whether it was a boy or girl?

29. If you had a miscarriage/lost your child during pregnancy or after birth, how did it affect you?

- How did your family treat you?
- Did the community treat you/the family differently? (e.g. isolation etc.)

*Exploring a future intervention*

30. Who would you trust to talk about problems in your pregnancy and after the birth?

- E.g. a village elder/woman
- A group of women who share the same experience?
- A doctor?

31. Do you meet up regularly (e.g. once a week) with other women to talk about your thoughts and feelings during the pregnancy? And after birth?

32. Today, do you have a phone for yourself (not shared use with husband)? Do you use it?

- How would you feel to receive text messages about pregnancy and taking care of mental health?

33. Would you find it helpful to have support for mental health during pregnancy

- To learn how to take care of good mental health?
- How to manage stress and negative thoughts?

***Conclusion***

Thank you very much for taking the time to answer our questions and for taking part in the PRAMH study. Is there anything you would like to ask or say?

If anything in our discussion made you uncomfortable or has unsettled you, please do come forward and speak to me and my colleagues. We will be very happy to talk to you about your experiences.

**Additional file 1.2: Topic guide for focus group discussions with ASHAs/AMNs**

***Overview***

This is the topic guide for interviews. The questions below are prompts to explore details on perinatal mental health in rural India. The questions should be seen as prompt for triggering discussions and conversations on topic areas, rather than attempting to cover all points listed.

***Introduction***

Thank you for taking part in the PRAMH study and this focus group discussion.

The wider research aim of the PRAMH study is to understand the context and state of perinatal mental health systems, services, and resources in India, specifically in the states of Haryana and Telangana.

To investigate this and understand the context and details of perinatal mental health in rural areas of Haryana and Telangana, we have invited you as experts to discuss relevant topics and help answer our questions.

The focus group discussion will explore a number of topics around perinatal mental health and people living with mental health problems, how they seek and receive help, cultural values and norms, barriers to get help, and stigma and discrimination.

The focus group discussion should take no longer than 1.5 hours. You are free to pause, stop, ask questions, and withdraw from our conversation at any time, without giving reason and without any consequences for yourself.

***List of the topic areas and examples of prompts/questions that could be explored:***

*Intro & context*

1. Can you tell us about your home and where you live?

2. How does your usual day look like?

*General mental health knowledge, attitudes/perception and behaviour*

1. Are there people with mental health problems in your village?

2. Did you ever see a woman with mental health problems in pregnancy/after birth?

- Clarify that some mental health problems are more extreme (severe mental disorders/psychosis) and some are not that extreme/visible (common mental disorders).
- Mental illness may be understood as: Physical appearance: roaming on the road, dirty
- Mental illness may mean that people are feeling very low/depressed, or very anxious/worried or suicidal thoughts.

3. How do you feel when you see someone suffering from their mental health?

4. What are the reasons you think that people are having mental health problems?

- E.g. Because of illness, because of something in the past (previous birth), having a girl child, exhaustion, household responsibilities, family problems, etc.

5. How do you feel when they come to see you in the clinic?

6. What do people in your village think and say about women who are pregnant/one year after birth with mental health problems (anxiety/depression/suicidal thoughts)?

7. How do people in your community/village treat/take care of these women?

*Women with mental health problems in pregnancy and after childbirth*

8. What are the most common mental health problems in pregnant women/mothers?

9. Do you know how the pregnant women/mothers with mental health problems take care of themselves?

- Do they use kollu/alcohol to cope?

10. Where do pregnant women/mothers with mental health problems go to get help?

- What treatments/therapies are available? ASHA, family, spiritual healer, etc.?

11. What challenges are there when she would like to get help for mental health?

12. If a woman feels low/depressed or very anxious/worried, this can affect her physical health and that of her baby. Would this be a good reason to seek help for the negative feelings?

13. Would the husband and family support a woman if she needs help for mental health?

- And if this would help the baby?

14. How could the family/community help the women with mental health problems?

15. When women during pregnancy and after birth experience a lot of distress, they might think of taking their own life. Have you ever seen a woman in such a situation who wanted to die?

- Do you feel it is OK to talk about suicidal thoughts/suicide during pregnancy and after birth?
- If a woman in pregnancy or after birth is thinking of taking her own life, what will family/community around her do? What do you feel they should do?

*Care for women with perinatal mental health problems*

16. How much time do you usually spend with a woman who is pregnant and/or within one year that they have given birth?

17. How many women who are pregnant/one year after birth with mental health problems (anxiety/depression/suicidal thoughts) do you seen on average (per month/year)?

18. Did you ever receive any training in what mental health and mental disorders are?

- And specifically, for women who are pregnant/have given birth?

19. When a woman has mental health problems, what do you do?

- E.g. Talk, ask about family, husband, etc.
- Referral to GP/ specialist mental health care
- AYUSH doctors, spiritual healer
- Do you talk to her family (husband/mother in law) about this?

20. Do you know who is the mental health care expert in the district for perinatal women?

21. Do you know of any medications that you would give women who are pregnant/have given birth if they have mental health problems?

22. Do you know of any other therapies/treatments?

23. Do you record it when a woman who is pregnant/has given birth has a mental health problem?

24. Do you see any women with HIV?

- And that have mental health problems?

25. Have you seen women who are pregnant/have given birth who drink alcohol/the village drink (Kollu) often/habitually?

26. Is there any district/state mental health programme for women during pregnancy/after birth that you are aware of?

*Exploring a future intervention*

27. Who would be the best person/group where women can go to for help?

28. Would you be interested in talking to women with mental health problems in

pregnancy/after birth?

- Do you think it would be helpful to give them support for mental health during pregnancy to learn how to take care of good mental health, how to manage stress and negative thoughts?
- How much time do you have with a woman? Could the intervention be tagged on to their work?
- Who else in the village would be a respected person to carry out an intervention? E.g. a researcher/clinician, a community health worker, a village elder/woman, etc?

29. Would it be more acceptable to women if they were seeing the ASHA and they explained to them what common mental disorders are and how they can affect other (existing) health conditions, such as hypertension, diabetes, anaemia, or HIV, TB?

30. Do women meet up regularly (e.g. once a week) with other women during the pregnancy? And after birth?

- What would be a good place &amp; context if women were to receive a mental health intervention?
- Would there be any barriers to women attending a weekly session? E.g. transportation, employment, family responsibilities and child care

31. Do the women have and use a phone for yourself (not shared use with husband)?

32. Do you think a shared experience/group intervention would be better or better to deliver the intervention to women individually?

***Conclusion***

Thank you very much for taking the time to answer our questions and for taking part in the PRAMH study. Is there anything you would like to ask or say?

**Additional file 1.3: Topic guide for focus group discussions with families & carers**

***Overview***

This is the topic guide for interviews. The questions below are prompts to explore details on perinatal mental health in rural India. The questions should be seen as prompt for triggering discussions and conversations on topic areas, rather than attempting to cover all points listed.

***Introduction***

Thank you for taking part in the PRAMH study and this focus group discussion.

The wider research aim of the PRAMH study is to understand the context and state of perinatal mental health systems, services, and resources in India, specifically in the states of Haryana and Telangana.

To investigate this and understand the context and details of perinatal mental health in rural areas of Haryana and Telangana, we have invited you as experts to discuss relevant topics and help answer our questions.

The focus group discussion will explore a number of topics around perinatal mental health and people living with mental health problems, how they seek and receive help, cultural values and norms, barriers to get help, and stigma and discrimination.

The focus group discussion should take no longer than 1.5 hours. You are free to pause, stop, ask questions, and withdraw from our conversation at any time, without giving reason and without any consequences for yourself.

***List of the topic areas and examples of prompts/questions that could be explored:***

*Intro & context*

1. Can you tell us about your home and where you live?

2. How does your usual day look like?

*General mental health knowledge, attitudes/perception and behaviour*

3. Did you ever see anyone suffering from mental illness?

4. Some mental health problems are not as extreme, it may mean that people are feeling very low/depressed, or very anxious/worried. Have you met people with these problems?

- Mental illness may be understood as: Physical appearance: roaming on the road, dirty = severe MD. But we are talking about CMDs
- Perception that depression, anxiety is not the same mental disorders/ suicidal thoughts

5. How do you feel when you see someone suffering from their mental health?

- Would you work with them/ live with them? Be friends with them? Be married to them?

6. What are the reasons you think that people are having mental health problems?

- E.g. Because of illness, because of something in the past (previous birth), having a girl child, exhaustion, household responsibilities, family problems, etc.

7. What do people in your village think and say about women who are pregnant/one year after birth with mental health problems (anxiety/depression/suicidal thoughts)?

8. How do people in your community/village take care of someone with a mental health problem?

9. Would you take care of someone if they had a mental health problem? Within your family (how close?)

*Women with mental health problems in pregnancy and after childbirth*

10. What are the most common mental health problems in pregnant women/mothers?

11. Do you know how the pregnant women/mothers with mental health problems take care of themselves?

- Do they use kollu/alcohol to cope?

12. Where do pregnant women/mothers with mental health problems go to get help?

- What treatments/therapies are available? ASHA, family, spiritual healer, etc.?

13. What challenges are there when she would like to get help for mental health?

14. How could the family/community help the women with mental health problems?

15. When women during pregnancy and after birth experience a lot of distress, they might think of taking their own life. Have you ever seen a woman in such a situation who wanted to die?

- Do you feel it is OK to talk about suicidal thoughts/suicide during pregnancy and after birth?
- If a woman in pregnancy or after birth is thinking of taking her own life, what will family/community around her do? What do you feel they should do?

16. Have you seen women who are pregnant/have given birth who drink alcohol/the village drink (Kollu) often/habitually?

- And/or drugs?

*Care for women with perinatal mental health problems*

17. Have you ever cared for a woman who is pregnant and/or within one year that they have

given birth?

- What did you do?
- How much time did you usually spend with her?

18. What kind of problem did the woman have?

- E.g. Anxious, depressed, suicidal
- Birth of undesired gender
- Miscarriage/ death of infant

19. What were your thoughts and feelings about her being sick/unwell?

- Why do you think she got unwell?
- Did her illness make you feel different about her?

20. How was your experience to care for her?

- Did you find it ok/ challenging/ embarrassing, etc.?
- Time effort, money, logistics, etc.

21. Did you ever receive any training in what mental health and mental disorders are?

- And specifically, for women who are pregnant/have given birth?

22. Did/do you get help or advice from anyone how to take care of the woman?

- E.g. ASHA/ psychiatrist, etc.

23. Do you know of any medications that you would give women who are pregnant/have given birth if they have mental health problems?

- Do you know of any other therapies/treatments?

*Exploring a future intervention*

24. Do you think it would be helpful to give the woman support for mental health during pregnancy to learn how to take care of good mental health, how to manage stress and negative thoughts?

25. If a woman feels low/depressed or very anxious/worried, this can affect her physical health and that of her baby (such as hypertension, diabetes, anaemia, or HIV, TB). Would this be a good reason to seek help for the negative feelings?

26. Would the husband and family support a woman if she needs help for mental health?

- And if this would help the baby?

27. Who would be the best person/group where women during pregnancy and after the birth can go to for help?

- Who in the village would be a respected person to carry out an intervention? E.g. a researcher/clinician, a community health worker, a village elder/woman, etc?

28. Do you think the woman could go out to meet other women/ get some support once a week?

- Would there be any barriers to the women attending a weekly session? E.g. transportation, employment, family responsibilities and child care?

29. Would it be helpful for you to learn more about what mental health problems are and how to care for a woman who is pregnant or with a new baby with mental health problems?

***Conclusion***

Thank you very much for taking the time to answer our questions and for taking part in the PRAMH study. Is there anything you would like to ask or say?

If anything in our discussion made you uncomfortable or has unsettled you, please do come forward and speak to me and my colleagues. We will be very happy to talk to you about your experiences.

**Additional file 1.4: Topic guide for in-depth interviews**

***Overview***

This is the topic guide for interviews. The questions below are prompts to explore details on perinatal mental health in rural India. The questions should be seen as prompt for triggering discussions and conversations on topic areas, rather than attempting to cover all points listed.

***Introduction***

Thank you for taking part in this interview for the SMARThealth Pregnancy and Mental Health (PRAMH) study. The wider research aim of the PRAMH study is to understand the context and state of perinatal mental health systems, services, and resources in India, specifically in Telangana.

In this interview, I will ask you a few questions to understand the details of perinatal mental health in rural areas of Telangana. The questions will explore a number of topics around perinatal mental health, for instance around formal and informal health and care systems and services, policies and legislation, or the social and political processes, cultural values and norms, and factors, barriers and facilitators, including stigma and discrimination.

The interview should take no longer than one hour. You are free to pause, ask questions, stop and withdraw from our conversation at any time, without giving reason and without any consequences for yourself.

***List of the topic areas and examples of prompts/questions that could be explored:***

*Intro & context*

1. Can you tell me about yourself and your role?

*Perinatal mental health politics, policies and plans*

2. What perinatal mental health policies, legislation, and plans are in place?

- Is there a perinatal mental health budget?
- How do these plans, etc. address equity?

3. What is the political support for perinatal mental health?

4. What do you know in terms of perinatal mental health professionals/human resources?

*Perinatal mental health treatment coverage*

1. What are the most common mental health problems in women/ mothers?

2. What is the prevalence of perinatal mental disorders?

3. What was the service coverage of women with perinatal mental disorders in the past year?

4. What was the treatment coverage of women with perinatal mental disorders in the past year?

5. What were the referral rates to specialist mental health care?

*District level health services for Perinatal Mental health (PMH)*

6. How are the district mental health services organized for perinatal mental health?

7. What are the administrative structures for PMH in the district?

8. What are the available human resources in specialist and general care?

9. What are the available in-patient care services?

10. What maternal/perinatal health care facilities &amp; resources are there?

11. What mental health care is provided in the district for perinatal women?

12. What HIV mental health care is provided in the district for perinatal women?

13. What mental health care for PMH is there in PHC?

14. What mental health training for perinatal women is provided in the district in PHC/specialist care?

15. What is the essential psychotropic medication availability for PMH in PHC?

16. What is the essential psychotropic medication availability for PMH in the nearest health post?

17. How is financing and provision of psychotropic medication organised?

18. What psychosocial interventions are available for PMH?

19. What are the PHC/ perinatal/ mental health service/ community interfaces?

20. What non-health sector activities are relevant to perinatal mental health?

21. Is there any district/state mental health programme for women during pregnancy/after birth that you are aware of?

22. What (perinatal) mental health promotion / mental disorder prevention / awareness-raising/ anti-stigma activities were/are done within the district?

*Monitoring and evaluation*

23. What general health information systems exist for perinatal mental health?

24. How is perinatal mental health surveillance information recorded and reported?

25. What indicators are being measured?

26. How is the quality of mental health care monitored?

*Women with mental health problems in pregnancy and after childbirth*

27. What are the most common mental health problems in pregnant women/mothers?

28. How can we best capture mental health? What is the thinking/words of mental health?

29. What do people in think and say about women who are pregnant/one year after birth with mental health problems (anxiety/depression/suicidal thoughts)?

30. How do people talk about suicidal thoughts/suicide in women in pregnancy/after birth?

31. Are you aware of any stigma/discrimination towards women with mental health problems in pregnancy/after birth?

- What kinds of stigma/discrimination?

32. How do people in the community/village treat/take care of women during pregnancy/after birth with mental health problems?

33. Would the husband and family support a woman if she needs help for mental health?

34. Do you know how the pregnant women/mothers with mental health problems take care of themselves?

- Do they use kollu/alcohol to cope?

35. Where do pregnant women/mothers with mental health problems go to get help?

- What treatments/therapies are available? ASHA, family, spiritual healer, etc.?

36. What challenges are there when she would like to get help for mental health?

37. How could the family/community help the women with mental health problems?

38. If a woman feels low/depressed or very anxious/worried, this can affect her physical health and that of her baby. Would this be a good reason to seek help for the negative feelings?

*Exploring a future intervention*

39. Do you think it would be helpful to give women support for mental health during pregnancy to learn how to take care of good mental health, how to manage stress and negative thoughts?

40. Would it be more acceptable to women if they were seeing the ASHA and they explained to them what common mental disorders are and how they can affect other (existing) health conditions, such as hypertension, diabetes, anaemia, or HIV, TB?

41. Do you think the intervention would be accepted by the husband and family if it would be benefitting the baby as well?

42. Which type of intervention would women find most useful and acceptable?

- E.g. stress management, breathing, control of thoughts, emotions and behaviors, problem solving, self-care techniques, psycho-education, positive activity scheduling (behavioural activation), etc?

43. Who would be the best person or group where women can go to for help? Who in the village would be a respected person to carry out an intervention?

- E.g. a researcher/clinician, a community health worker, a village elder/woman, etc?
- Could the intervention be tagged on to the ASHAs’ work?

44. Do you think a shared experience/group intervention would be better or better to deliver the intervention to women individually?

45. Do you know of any scales that have been used to assess women’s mental health during pregnancy/after birth?

- Such as GHQ12, GAD7, EDPS

46. Who are other important people we should talk to?

***Conclusion***

Thank you very much for taking the time to answer my questions and for taking part in the PRAMH study. Is there anything you would like to ask or say?
