# Supplementary material for: Perinatal mental health and its social determinants: qualitative findings from rural Telangana
Source: BMC Psychiatry. 2026 May 23;26:553. doi: 10.1186/s12888-026-08066-1 (PMC13390382; doi:10.1186/s12888-026-08066-1)
Supplement: Supplementary file 2 — Supplementary Material 2 [file 12888_2026_8066_MOESM2_ESM.docx]

# **Additional file 3: Definitions and distribution of the social determinants across the five major themes, with illustrative quotations**

*Abbreviations*: CMD = common mental disorder, ASHA = Accredited Social Health Activist, AWW = Anganwadi worker, AYUSH = Ayurveda, Yoga & Naturopathy, Unani, Siddha, and Homeopathy, FGD = focus group discussion, IDI = in-depth interview

**Additional file 3.1: Theme 1 - Attitudes about pregnancy outcomes**

| **Socio-ecological level** | **Social determinant** | **Definition** | **Illustrative quotations** |
| --- | --- | --- | --- |
| **Society** | Son preference/ daughter aversion | Societal norms that favor the birth of male children & disfavor the birth of female children | *“We need son as he is the heir. Daughter will get married away to some other family. I have two granddaughters and I need grandson very badly”* ***(Caregivers, FGD6).*** |
|  | Discrimination & exclusion | Prejudiced treatment of individuals based on gender, pregnancy outcome, or mental health status | *“[Some] will not allow other pregnant woman to go near or meet [women that have had a pregnancy loss]. Do they [do] the same in your village?”* ***(Interviewer).***  *“Yes… She had a [pregnancy loss], so this girl might also get that.”* ***(Caregiver, FGD7)****.* |
|  | Cultural & faith-based practices | Values, beliefs, and traditions that shape perinatal women’s experiences & mental health | *“God is there. He will bless you with another baby. God might have taken away as he gave you this [pregnancy loss]”* ***(Caregiver, FGD6)****.* |
|  | Attitudes about pregnancy outcomes | Views on fertility, pregnancy loss, infant health and gender outcomes that affect perinatal mental health | *“If the mind of the mother is fresh (worry free), the growth of the baby will be much better. All her tension will be reflected in the weight of the baby”* ***(AWW, FGD3)****.* |
|  | Women’s roles & responsibilities | Behaviors & duties that women are expected to fulfill in their households, communities, and societies | *“There's this increased desire of the families to have a male child… [If all children] turns out to be female, the woman feels inadequate that she's not able to give successor to the family”* ***(Psychiatrist, IDI8).*** |
|  | Gender equity | The distribution of opportunities, resources, and status between genders that permits equal consideration of their respective needs & well-being | *“With the pressure [to produce] a heir to the family, [there] are cases where women [have] attempted suicide… I saw a case of an attempted infanticide [because] the fourth child was also a daughter”* ***(Psychiatrist, IDI8)****.* |
| **Interpersonal relationships** | Infant gender outcome | Expectations & responses to gender outcomes from pregnancies that impact perinatal mental health | *“[Women] are worried if they deliver the baby girl the in-laws might not treat her well. If she delivers a baby girl, [the in-laws] will not care about her. If she delivers a baby boy they celebrate her”* ***(Caregiver, FGD6)****.* |
|  | Gender of previous children | The gender composition of women’s existing children as it impacts their mental health during subsequent pregnancies | *“They [think] ‘I have given birth to 2 girl children, if the son is not born now… my family members will not accept’”* ***(ASHA, FGD4).*** |
|  | Pregnancy/ infant health outcome | Expectations, fears, and responses to infant health and pregnancy outcomes that impact perinatal mental health | *“She started feeling guilty about it [and thought] that because of my condition, my marriage, this child has become premature”* ***(Clinician, IDI1)****.* |
|  |  |  | *“If the mother has some infection or the baby has some infection, the pain is borne by the mother”* ***(Women at risk for perinatal CMDs, FGD9)****.* |
|  | Domestic violence | Physical, verbal, and emotional abuse and neglect from women’s partners and family members | *“If it is [a] boy, they are [enthusiastic]. If it is girl… they will manhandle the wives. Even in-laws torture the lady for boy child”* ***(Women at risk for perinatal CMDs, FGD9)****.* |
|  |  |  | *“If you do not have a baby girl, you cannot stay in the house. This is what the husband says… [She] is in her 5^th^ pregnancy… She is tensed up”* ***(AWW, FGD3).*** |
| **Individual** | Previous pregnancy loss | Expectations & experiences from prior miscarriages, abortions, and adverse pregnancy outcomes that impact perinatal mental health during subsequent pregnancies | *“Those who have experienced complications in first pregnancy will have a fear that there might be complications in the second pregnancy also”* ***(AYUSH doctor, IDI3)****.* |
|  | Number of previous pregnancies | Women’s primiparous or multiparous status as it influences their perinatal mental health | *“She was tensed during her first child that the delivery has to be good”* ***(Caregiver, FGD7).*** |
|  | Delivery outcome | Women’s expectations, fears, and understandings of different delivery modalities (cesarean section, vaginal birth, etc.) |  |
|  | Maternal health conditions | Gestational and pre-gestational health conditions that impact women’s perinatal mental health | *“In the case I have seen, if the lady already has [diabetes], there will be a chance of [pregnancy loss]. They will start feeling ‘I am not able to get a child.’ Even [in] high blood pressure cases, they will feel ‘Will my blood pressure come down or not, how will my delivery happen?’” (****ASHA, FGD5).*** |

**Additional file 3.2: Theme 2 – Gender inequity**

| **Socio-ecological level** | **Social determinant** | **Definition** | **Illustrative quotations** |
| --- | --- | --- | --- |
| **Society** | Women’s roles & responsibilities | Behaviors and duties that women are expected to fulfill in their households, communities, and societies | *“You go to work, come back. Do you feel like all the… house[work] like washing clothes, cooking [must be] done by you?”* ***(Interviewer).***  *“[Yes], we cry [before] we start doing the work”* ***(Women at risk for perinatal CMDs, FGD9).*** |
|  |  |  | *“I was unable to do chores. He left me based on his mother’s words”* ***(Women at risk for perinatal CMDs, FGD9).*** |
|  | Gender equity | The distribution of opportunities, resources, and status between genders that permits equal consideration of their needs and well-being | *“So the concept of empowerment is not there. Concept of even you making, thinking of your own thoughts, your own sense of what are my feelings, what are my emotions, what are my thoughts is not going to work… There is nothing called that concept in our Indian culture. The minute you say that I, the word I comes in, you're out of the family… The concept of her individuation her empowerment, her agency is not there”* ***(Clinician, IDI1).*** |
|  | Attitudes about domestic violence | Understandings and perceptions of physical, verbal, and emotional abuse that women experience from partners and family members | *“They do often discuss [with] other women… But again, it's all about hushing themselves and like, you know, trying to adjust to the whole societal stereotypical views… It's rare to hear women actually encourage other[s] to stand up for themselves… [they usually encourage women] to try to maintain the toxic relationship [as] they feel breaking a relationship is more traumatic than undergoing [domestic violence]”* ***(Psychiatrist, IDI8).*** |
| **Policy** | Nutritional services | Local and national programs targeting maternal malnutrition, and their impact on perinatal mental health | *“Mother-in-law will not allow the daughter-in-law to eat the food served in Anganwadi… They will ask ‘if egg is given daily, only you will [get to] eat it daily is it?”* ***(AWW, FGD2)****.* |
| **Community** | Nutritional access | Diet quality and accessibility, and its impact on women’s physical and mental health during the perinatal period | *“Some [husbands] say ‘you should eat only after we eat, how can you eat before serving us’… It is very rare that such people are not there”* ***(AWW, FGD2).*** |
|  | Employment conditions & nature | Demands and responsibilities in the workplace that impact women’s perinatal mental health | *“One girl had lit herself using kerosene [because when she got pregnant], her husband and mother-in-law came together and they sent her out saying ‘I don’t want the child’… She [now] lives alone with two children”* ***(ASHA, FGD5).***  *“How about the earnings?”* ***(Interviewer).***  *“She works and [makes a] living. Even during pregnancy she was working… She is taking care of both children and living… but she keeps crying. She cries a lot”* ***(ASHA, FGD5).*** |
|  | Financial well-being | The ability for women and their families to meet their financial needs, and the impact of poverty on perinatal mental health | *“If husband is alcoholic, kids responsibilities are only given to single person [and] we suffer to bring the groceries home”* ***(Caregivers, FGD6).*** |
| **Interpersonal relationships** | Alcohol use by husband | How alcohol use by husbands affects the support that women receive, responsibilities they hold, and mental health during the perinatal period |  |
|  | Involvement in pregnancy planning | Women’s role and autonomy in determining when, how, and how often conception occurs | *“Happens (conceives) without planning without her wish. Are there such cases?”* ***(Interviewer).***  *“Yes, many such cases will be there… There are people who have given birth to two children within one year”* ***(ASHA, FGD5).***  *“Will they be stressed?”* ***(Interviewer).***  *“Yes, very much. They will not be able to eat, can’t take care of the child”* ***(ASHA, FGD5).*** |
|  | Pregnancy/infant health outcome | Expectations, fears, and responses to infant health and pregnancy outcomes that impact perinatal mental health | *“She fears [miscarriage] but mother-in-law will say ‘when we were pregnant we used to do all the chores’”* ***(Caregiver, FGD7)****.* |
|  | Relationship & support from in-laws | The nature and quality of connection that women have with their in-laws, as it impacts their mental health and support during the perinatal period | *“The mother-in-law would… enter into their physical spaces telling her when she should have sex and when she should not have sex… When she was pregnant, [mother-in-law] would eat the whole food and not keep anything for her”* ***(Clinician, IDI1)****.* |
|  | Relationship & support from husband | The nature and quality of connection that women have with their partners, as it impacts their mental health and support during the perinatal period | *“She retained the 4th pregnancy… because her husband was forcing saying… ‘I want a son’… She was not able to oppose the husband… She became pregnant back-to-back very fast”* ***(ASHA, FGD5)****.* |
|  | Domestic violence | Physical, verbal, and emotional abuse and neglect from women’s partners and family members | *“Society may not allow collective action [against domestic violence] because violence is not to be spoken about”* ***(Clinician, IDI1).*** |
|  |  |  | *“Women are to be beaten if they don't cook properly, if the salt is not okay, if the X, Y, Z is not okay, it is okay for the woman to be beaten up”* ***(Clinician, IDI1).*** |
| **Individual** | Maternal health conditions | Gestational and pre-gestational health conditions that impact women’s perinatal mental health | *“During pregnancy, the lady has a lot of pain…You said nobody cares. If they are in so much pain, it will affect pregnancy. They might have physical problems, like increase in [blood pressure], diabetes. If they know that such problem might be there, will [family members] take concern then?”* ***(Interviewer).***  *“Initially they never care. Later when someone else… talks [to them], then they might listen. If she herself says that they will never listen. They think that it is common.* ***(Women at risk for perinatal CMDs, FGD9).*** |

**Additional file 3.3: Theme 3 – Relationships and support**

| **Socio-ecological level** | **Social determinant** | **Definition** | **Illustrative quotations** |
| --- | --- | --- | --- |
| **Society** | Mental health stigma | Negative attitudes and stereotypes held against individuals experiencing perinatal CMDs | *“When you take her [for mental healthcare] do you think [that] the neighbors may think badly about your daughter?”* ***(Interviewer).***  *“My daughter [must] be healthy and happy. I don’t care about others”* ***(Caregiver, FGD6).*** |
|  | Son preference/ daughter aversion | Societal norms that favor the birth of male children & disfavor the birth of female children | *“Will parents put the pressure [to have a son] on their daughter? Only in-laws do that?”* ***(Interviewer).***  *“As they are same blood, [women’s parents] won’t such things to their daughters” (****Caregiver, FGD6).*** |
|  | Attitudes about pregnancy outcomes | Views on fertility, pregnancy loss, infant health and gender outcomes that affect perinatal mental health | *“So it was aborted due to bad heartbeat. Did you stay in the hospital?”* ***(Interviewer).***  *“After[wards] for one month I took bed rest”* ***(Women at risk for perinatal CMDs, FGD9).***  *“You took bed rest. Your family, neighbors, did they support you or taunt you”* ***(Interviewer).***  *“I was at my mother’s place. My mother took care of me.* ***(Women at risk for perinatal CMDs, FGD9).*** |
|  | Cultural & faith-based practices | Values, beliefs, and traditions that shape perinatal women’s experiences & mental health | *“There will baby shower at 9 month. We have to offer new clothes; we have to invite the whole family”* ***(Caregiver, FGD6).***  *“Will she be in anxiety at that time?”* ***(Interviewer).***  *“Yes. About the formalities… We have to offer clothes, have to invite their relatives, have to cook five types of food”* ***(Caregiver, FGD6).***  *“Do you invite the villagers for lunch?”* ***(Interviewer).***  *“Yeah, we do”* ***(Caregiver, FGD6).***  *“How much will you spend for that?”* ***(Interviewer).***  *“It will cost around 10-20 thousand [rupees]”* ***(Caregiver, FGD6).***  *“So, will your daughter say to not celebrate it [in a] grand manner and to keep it low?”* ***(Interviewer).***  *“Yeah, sure she says, but will son listen to that [if] the daughter says it”* ***(Caregiver, FGD6).*** |
|  | Gender equity | The distribution of opportunities, resources, and status between genders that permits equal consideration of their respective needs & well-being |  |
| **Community** | Financial well-being | The ability for women and their families to meet their financial needs, and the impact of poverty on perinatal mental health | *“I don’t even have a hut… [but] we need good health of our children, we have to go [to private care]”* ***(Caregiver, FGD7).*** |
|  | Community relationships | The quality and quantity of connection that women have with others in their communities, as it impacts perinatal mental health | *“The neighbors also visit to know her wellbeing, they will also give her strength by telling her [that] nothing has happened and everything will be fine”* ***(Caregiver, FGD6).*** |
|  | Nutritional access | Diet quality and accessibility, and its impact on women’s physical and mental health during the perinatal period | *“Her in-laws do not treat her properly. When she was pregnant, she never [got] vegetables at home. When they were not seeing my mother and others used to give her… vegetables to eat”* ***(Women at risk for CMDs, FGD9)****.* |
| **Interpersonal relationships** | Peer relationships & support | The quality and quantity of connection that women have with friends and neighbors, as it impacts their mental health and support during the perinatal period |  |
|  | Relationship & support from in-laws | The nature and quality of connection that women have with their in-laws, as it impacts their mental health and support during the perinatal period | *“A few people look after the wife when she gets pregnant, but a few do not bother to do that. They don’t care. I have 3 boys and my husband does [not] even lift a vessel of water. My mother-in-law does not care”* ***(Women at risk for perinatal CMDs, FGD9).*** |
|  |  |  | *“Your daughter-in-law might have… mental disturbance during [pregnancy]… Will [your son] oppose her [going to mental healthcare]?”* ***(Interviewer).***  *“If he says that we will help her… we will… make him understand [so] she will be happy”* ***(Caregiver, FGD6).*** |
|  | Relationship & support from husband | The nature and quality of connection that women have with their partners, as it impacts their mental health and support during the perinatal period | *“Some husbands are supportive, even though the mother-in-law and father-in-law trouble them… In some cases husband will not be supportive but in-laws will be”* ***(ASHA, FGD4).*** |
|  |  |  | *“I was very anemic… I had a lot of tension… [My husband] supported a lot. He was the one who got the blood for me. I told him, ‘You get it tested multiple times because if [the donor has] any disease, I might get that disease [and] people will feel I have done some mistake…’ He got everything done”* ***(Self-help group leader, IDI5).*** |
|  |  |  | “*[My husband] used to listen to his mother and sister and did nothing to help. [After ASHAs said] that is not okay, he… helps a little”* ***(Women at risk for perinatal CMDs, FGD9)****.* |
|  | Relationship & support from own family | The nature and quality of connection that women have with their own parents, siblings, aunts/uncles, cousins, and more, as it impacts their mental health and support during the perinatal period | *“We also offer her palm milk…We buy a bottle for 100 rupees… if she asks we will bring it”* ***(Caregiver, FGD6).***  *“Wouldn’t that be expensive?”* ***(Interviewer).***  *“Our daughter should be happy”* ***(Caregiver, FGD6).*** |
|  | Alcohol use by husband | How alcohol use by husbands affects the support that women receive, responsibilities they hold, and mental health during the perinatal period | *“If he doesn’t work and 24 hours he keeps drinking… then [will she] have tension?”* ***(Interviewer).***  *“Yes. Husband should be good. If the husband says [no matter if it is] a boy or girl, we will take care, then wife will not feel bad. But if he goes on insisting on a boy baby, then she will become anxious and get mentally disturbed”* ***(Caregiver, FGD7).*** |
|  | Domestic violence | Physical, verbal, and emotional abuse and neglect from women’s partners and family members | *“If it is [a] boy, they are [enthusiastic]. If it is girl… they will manhandle the wives. Even in-laws torture the lady for boy child”* ***(Women at risk for perinatal CMDs, FGD9)****.* |
|  | Family structure | The organization of the women’s nuclear and extended family, including patrilocal customs that dictate who, when, and where they live with | *“First is at mother’s place. They take lot of care. Second is usually in in-laws place. But my mother-in-law never cared. So, my mother took me home for all the deliveries”* ***(Women at risk for perinatal CMDs, FGD9)****.* |
| **Individual** | Alcohol use during pregnancy | The impact that alcohol use by perinatal women has on their physical and mental health | *“Do [pregnant women] drink toddy (palm wine)? Will they ask you ‘madam can we drink toddy?’”* ***(Interviewer).***  *“Yes, if [pregnant women] ask we will tell them ‘no, don’t drink toddy, they will mix alcohol in it’ but they say that their family, their mother-in-law will not listen”* ***(ASHA, FGD5).***  *“Why is that so? Why will mother-in-laws ask them to drink toddy?”* ***(Interviewer).***  *“They feel that if [the woman] drinks toddy, the baby will be born fresh and neat”* ***(ASHA, FGD5).*** |

**Additional file 3.4: Theme 4 – Mental health stigma**

| **Socio-ecological level** | **Social determinant** | **Definition** | **Illustrative quotations** |
| --- | --- | --- | --- |
| **Society** | Mental health stigma | Negative attitudes and stereotypes held against individuals experiencing perinatal CMDs | *“When you [find] a bride for your son and hear that she has some mental illness like being sad and not opening up to anyone, and staying alone [will you accept?]”* ***(Interviewer).***  *“If she has such problems we don’t accept her… She doesn’t speak her heart so she might be careless.”* ***(Caregiver, FGD6).*** |
|  | Discrimination & exclusion | Prejudiced treatment of individuals based on gender, pregnancy outcome, or mental health status | *“How do villagers look at such people [with mental health problems]?”* ***(Interviewer)***  *“No one cares for them. They feel she doesn’t listen even if we speak. They will say ‘she is like that itself’” (****ASHA, FGD5).*** |
|  | Women’s roles & responsibilities | Behaviors & duties that women are expected to fulfill in their households, communities, and societies | *“Suicide is considered as a sign of weakness… Rather than considering why she [attempted] suicide, women are told why she shouldn't commit suicide, that now she has a responsibility to take care of the child, to run the family… So the feelings of the woman are invalidated and they're not acknowledged… especially in pregnant women”* ***(Psychiatrist, IDI8).*** |
| **Policy** | Sakhi centers | The accessibility and usage of community domestic violence centers | *“Going to help (Sakhi) centre is labelled as having a very big problem whereas women [may go] for smaller problems also. [Due to] this fear, many people don’t really come out and [seek help]”* ***(Psychiatrist, IDI8)***. |
| **Community** | Community relationships | The quality and quantity of connection that women have with others in their communities, as it impacts perinatal mental health | *“If it is a person whom we know, people might not share things with them… there will be fear about ‘will she share it with my mother-in-law or my mother”, so they will not discuss the actual issue”* ***(Caregiver, FGD7)****.* |
|  | Awareness of perinatal mental disorders & services | Understandings, attitudes, and knowledge of perinatal mental disorders and services among local communities | *“[Women] have been conditioned [to think] it is common… to feel depressed [and] anxious during pregnancy. They're not able to express their views [because of this], the fear of being labelled as a person with mental illness, or the lack of awareness that such problems exist. So they come into acceptance, or rather… denial that they actually are suffering from any mental health problem”* ***(Psychiatrist, IDI8).*** |
| **Organizations** | Perinatal mental health service accessibility | Ease in which women can initiate, attend, and receive care for perinatal mental disorders | *“If someone comes [to your home] and talks about mental health problems will you be able to talk freely?”* ***(Interviewer).***  *“We cannot talk madam.* *Home is a place where neighbors come, in-laws are present”* ***(Women with perinatal CMDs, FGD9).*** |
|  | Provider knowledge of perinatal mental health conditions & services | Understandings, attitudes, and knowledge of perinatal mental disorders and services among local perinatal health providers | *“We don't have community counselors correct in India. We have only ANMs and ASHAs and ANMs and ASHAs come from the same patriarchal family that the woman comes from and so if you tell her something, the ANM and ASHA want to give you the same societal message. No different from the society, what the societal message is”* ***(Clinician, IDI1).*** |

**Additional file 3.5: Theme 5 – Mental healthcare access**

| **Socio-ecological level** | **Social determinant** | **Definition** | **Illustrative quotations** |
| --- | --- | --- | --- |
| **Society** | Traditional healers & remedies | Healers and practices that address perinatal CMDs through methods rooted in the cultural beliefs and traditions of the local community | *“You said that only medicines will not help mental illnesses… go [away]”* ***(Interviewer).***  *“We take them to ‘Dargahs’, ‘Temples’ and even tie special bands”* ***(Women at risk for perinatal CMDs, FGD9).*** |
|  | Cultural & faith-based practices | Values, beliefs, and traditions that shape perinatal women’s experiences & mental health |  |
|  | Mental health stigma | Negative attitudes and stereotypes held against individuals experiencing perinatal CMDs | *“People feel “she is not sleeping well or not eating well, might be affected by spirit.” They will take them to holy places like Dargah or temples”* ***(AWW, FGD2).*** |
|  | Attitudes about domestic violence | Understandings and perceptions of physical, verbal, and emotional abuse that women experience from partners and family members | *“These villages are all small, close and connected groups… So if the news of a person walking into a Sakhi center goes [out], it's whole magnified and it's shown to the society that okay, she's having issues with the family and they've been turbulence and the arguments… So with this fear, many people don't really come out and [seek help]”* ***(Psychiatrist, IDI8).*** |
| **Policy** | Sakhi centers | The accessibility and usage of community domestic violence centers | *“But in severe cases when there's like imminent threat to the life of the patient or she's having suicidal thoughts, the government also has some centers called Sakhi Kendra, Sakhi centers which are for protection and empowerment of women… The women can walk into these centers and then these people from the Sakhi centers they refer these people to us [for] a psychiatric consultation if they feel that the woman is depressed or if she's undergoing some behavioral abnormalities or any trauma”* ***(Psychiatrist, IDI8).*** |
|  | Inclusion of mental health in routine antenatal care | Status of perinatal mental health focus and care provision in routine maternal and child services | *“There's no proper screening test… for antenatal or postnatal women, especially in the rural parts. It's only when they are like observed… by the obstetrician, then they're brought to our notice… The gynecologist and obstetrician… or the [primary health center] doctors should be trained to screen people”* ***(Psychiatrist, IDI8).*** |
| **Organizations** | Perinatal mental health service accessibility | Ease in which women can initiate, attend, and receive care for perinatal mental disorders | *“Where will they be? Where will the psychiatrist be?”* ***(Interviewer).***  *“In [big city]”* ***(AWW, FGD1).***  *“Do they keep visiting your village?”* ***(Interviewer).***  *“No”* ***(AWW, FGD1).*** |
|  |  |  | *“So, there is nobody here to treat mental health problems of pregnant women… in your [primary health center]. How about District hospital?”* ***(Interviewer).***  *“[Psychiatrists] are there” (****AWW, FGD2).*** |
|  | Perinatal mental health service availability | The amount and quality of personnel and resources involved in perinatal mental healthcare | *“When I enquired here, there was no proper psychiatrist available. So, there was no chance that these people will go to the psychiatrist”* ***(Gynecologist, IDI4).*** |
|  |  |  | *“What are the available in-patient services [like]?”* ***(Interviewer).***  *“If I am not wrong, the government [district hospital] has 20 beds available”* ***(Medical officer, IDI7).*** |
|  | Provider knowledge of perinatal mental health conditions & services | Understandings, attitudes, and knowledge of perinatal mental disorders and services among local perinatal health providers | *“We were not trained in a special way. ASHAs and ANMs were also not given any special training. We gather the [antenatal care] cases along with ASHAs and ANMs. We, doctors ourselves will give counselling to those pregnant ladies”* ***(AYUSH doctor, IDI3)*** |
|  |  |  | *“So the ASHAs are the first contact point in most cases - how do you feel is the knowledge in ASHAs in relation to mental health problems during pregnancy and after birth?”* ***(Interviewer).***  *“The knowledge is not quite sufficient. I mean, they have little or meager knowledge about the perinatal mental health issues”* ***(Psychiatrist, IDI8)****.* |
